# Supplementary material for: TOE1 is a β-catenin interacting protein regulating the proliferation of hematopoietic cells through PAK2 modulation
Source: Stem Cell Reports. 2026 Apr 23;21(5):102894. doi: 10.1016/j.stemcr.2026.102894 (PMC13163219; doi:10.1016/j.stemcr.2026.102894)
Supplement: Document S1. Figures S1–S8 and Tables S2–S7 and supplementary methods [file mmc1.pdf]

Stem Cell Reports, Volume *21*

Supplemental Information

TOE1 is a β-catenin interacting protein regulating the proliferation of hematopoietic cells through PAK2 modulation

Hyun Park, Okan Sevim, Megan Wagstaff, Aaron Goff, David A. Palmer, Bomee Kim, Kate Heesom, Allison Blair, Sarah F. Newbury, Ethan L. Morgan, Benjamin P. Towler, Timothy J. Chevassut, and Rhys G. Morgan

Supplementary Table S2. Forward and reverse primer sequences utilised for RT-qPCR.

Oligo name	Sequence (5'→3')
GAPDH (Forward)	ACAGTCAGCCGCATCTTCTT
GAPDH (Reverse)	ACGACCAAATCCGTTGACTC
LEF1 (Forward)	AGAACACCCCGATGACGG
LEF1 (Reverse)	GGCATCATTATGTACCCGGAAT
PAK2 (Forward)	TGAGCACACCATCCATGTTGG
PAK2 (Reverse)	AGGTCTGTAGTAATCGAGCCC

Supplementary Table S3. Antibodies utilised for immunoblotting, immunofluorescence and flow cytometric assessment.

Antibody	Species	Manufacturer	Cat. number	Dilution
GAPDH	Mouse	Proteintech	60004-1-Ig	1:50,000
β-actin	Mouse	Merck Millipore	A1978	1:50,000
α-Tubulin	Mouse	Merck Millipore	T9026	1:50,000
Lamin A/C	Mouse	Merck Millipore	SAB4200236	1:50,000
β-catenin	Mouse	BD Biosciences	610154	1:2,000
TOE1	Rabbit	Bethyl	A303-643A	1:1,000
LEF-1	Rabbit	Cell Signalling Technology	2230S	1:1,000
TCF4	Rabbit	Cell Signalling Technology	2569S	1:1,000
TCF1	Rabbit	Cell Signalling Technology	2203S	1:1,000
PAK2	Rabbit	Cell Signalling Technology	2608S	1:1,000
PARN	Rabbit	Abcam	AB188333	1:1,000
CD34-PE	Mouse	BioLegend	343505	1:100
PE Mouse IgG1, κ Isotype Ctrl Antibody	Mouse	BioLegend	400111	1:100
CD45-PerCPCy5.5	Mouse	BioLegend	393409	1:100
PerCP/Cy5.5 Mouse IgG1, κ	Mouse	Biolegend	400149	1/100

Isotype Ctrl Antibody				
CD36-PE	Mouse	BioLegend	336205	1:100
PE Mouse IgG2a, κ Isotype Ctrl (FC) Antibody	Mouse	BioLegend	400213	1:100
CD13-PerCPCy5.5	Mouse	BioLegend	301713	1:100
PerCPCy5.5 IgG1 isotype control	Mouse	BioLegend	260114	1:100
PerCP/Cyanine5.5 Mouse IgG1, κ Isotype Ctrl	Mouse	BioLegend	400149	1:100
Alexa Fluor 647 anti-Rabbit IgG	Goat	Invitrogen	A-21245	1:500
Alexa Fluor 488 anti-Mouse IgG	Goat	Invitrogen	A-11001	1:500
anti-Mouse IgG (H+L) Secondary Antibody, HRP	Goat	Invitrogen	A4416	1:1000
anti-Rabbit IgG (H+L) Secondary Antibody, HRP	Goat	Invitrogen	A6154	1:1000

Supplementary Table S4. Lentiviral plasmid profiles utilised for transgene expression.

**Target gene**	**Expression type**	**Vector type**	**Source**
Non-targeting	shRNA control	pLKO.1-puro Non-Mammalian shRNA Control Plasmid SHC002	Merck Millipore
CTNNB1	shRNA#1	TRCN0000314920	Merck Millipore
CTNNB1	shRNA#2	TRCN0000314921	Merck Millipore
TOE1	shRNA#1	TRCN0000427713	Merck Millipore
TOE1	shRNA#2	TRCN0000152877	Merck Millipore
PAK2	shRNA#1	TRCN0000194671	Merck Millipore
PAK2	shRNA#2	TRCN0000002118	Merck Millipore
PAK2	shRNA#3	TRCN0000002116	Merck Millipore
PAK2	shRNA#4	TRCN0000002115	Merck Millipore
Non-targeting	shRNA control	pLV[shRNA]-EGFP-U6>Scramble (Vector Builder ID:VB230321-1431mhe)	Vector Builder
TOE1	shRNA	pLV[shRNA]-EGFPU6>hTOE1 (Vector Builder ID: VB240320-1219wyt)	Vector Builder
Empty vector control	Ectopic	pLV-EGFP-T2A-Puro-EF1A (Vector Builder ID: VB160723-1006snj)	Vector Builder
TOE1	Ectopic	pLV1-EF1A-hTOE1-Puro (Vector Bulder ID: VB250710-1214jbm)	Vector Builder
PAK2	Ectopic	pLV[Exp]-EGFP/Puro-EF1A>hPAK2 (Vector Builder ID: VB900173-3528fse)	Vector Builder

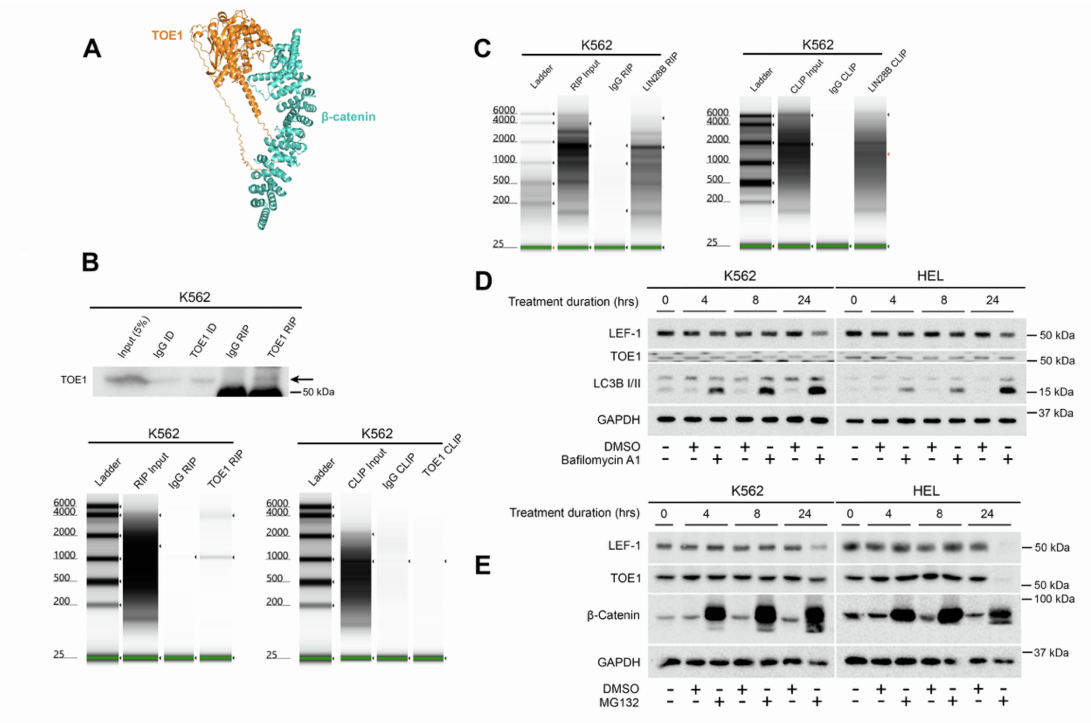

Supplemental Figure S1. β-Catenin forms an RNA-independent interaction with TOE1 in myeloid cells. **(A)** AlphaFold 3 prediction of the β-catenin:TOE1 interaction. The predicted template modelling (pTM) and interface PTM (iPTM) scores were low (0.46 and 0.41 respectively) (Evans et al., 2022; Jumper et al., 2021). To further study the macromolecular interface between the proteins, we utilised PDBePISA (Krissinel and Henrick, 2007). Although there was a considerable hydrophobic interface, as denoted by a solvation free energy gain on formation of the interface ($\Delta^{i}G$) score of -20.8, the complex formation significance score of 0.0 highlighted that the interface is unlikely to be critical for assembly formation. β-Catenin is represented in cyan, whilst TOE1 is represented in orange. **(B)** Immunoblot showing the enrichment of TOE1 in RIP samples above the IgG control. Non-specific bands above and below the TOE1 band (57 kDa) were observed in the co-IP analysis, with the specific TOE1 band represented by an arrow. TapeStation gel images showing the level of RNA isolated following TOE1 immunoprecipitation in RIP and CLIP assays. **(C)** TapeStation gel images demonstrating the enrichment of RNA isolated following LIN28B immunoprecipitation above IgG control in RIP and CLIP assays. Representative immunoblots demonstrating protein levels of LEF-1, β-catenin,

TOE1 and LC3B I/II in K562 and HEL cells ± **(D)** 1μM MG132 or **(E)** 100nM Bafilomycin A1. GAPDH was utilised as the loading control.

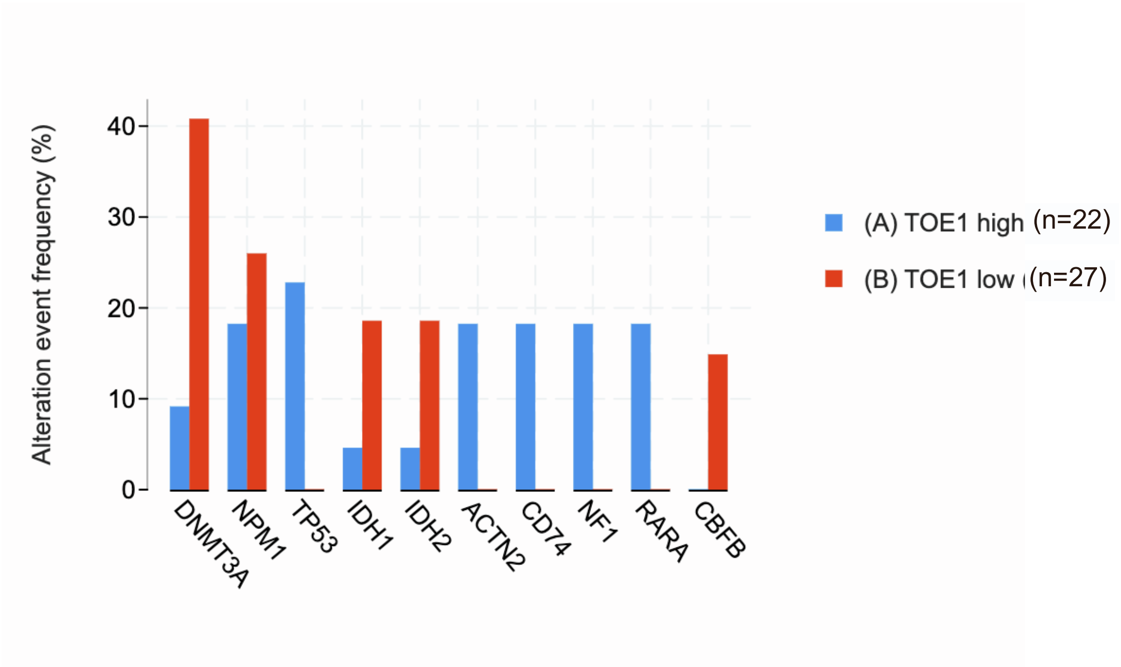

Supplemental Figure S2. *TP53* mutations are enriched in TOE1^{high} samples. Summary graph highlighting the top 10 most frequently altered genes in TOE1^{high} (z score ≥1; n=22) versus TOE1^{low} (z-score <1; n=27) mRNA samples, as derived from the New England Journal of Medicine (NEJM) 2013 The Cancer Genome Atlas (TCGA) acute myeloid leukemia whole exome sequencing data set.

Supplemental Table S5. Altered genes significantly enriched in TOE1^{high} AML patients as deduced from a two-sided Fished exact test.

Gene	Cytoband	% of patients with altered gene	p-Value
TP53	17p13.1	22.73% (5/22)	0.0138
ACTN2	1q43	18.18% (4/22)	0.0345
CD74	5q33.1	18.18% (4/22)	0.0345
NF1	17q11.2	18.18% (4/22)	0.0345

RARA	17q21.2	18.18% (4/22)	0.0345

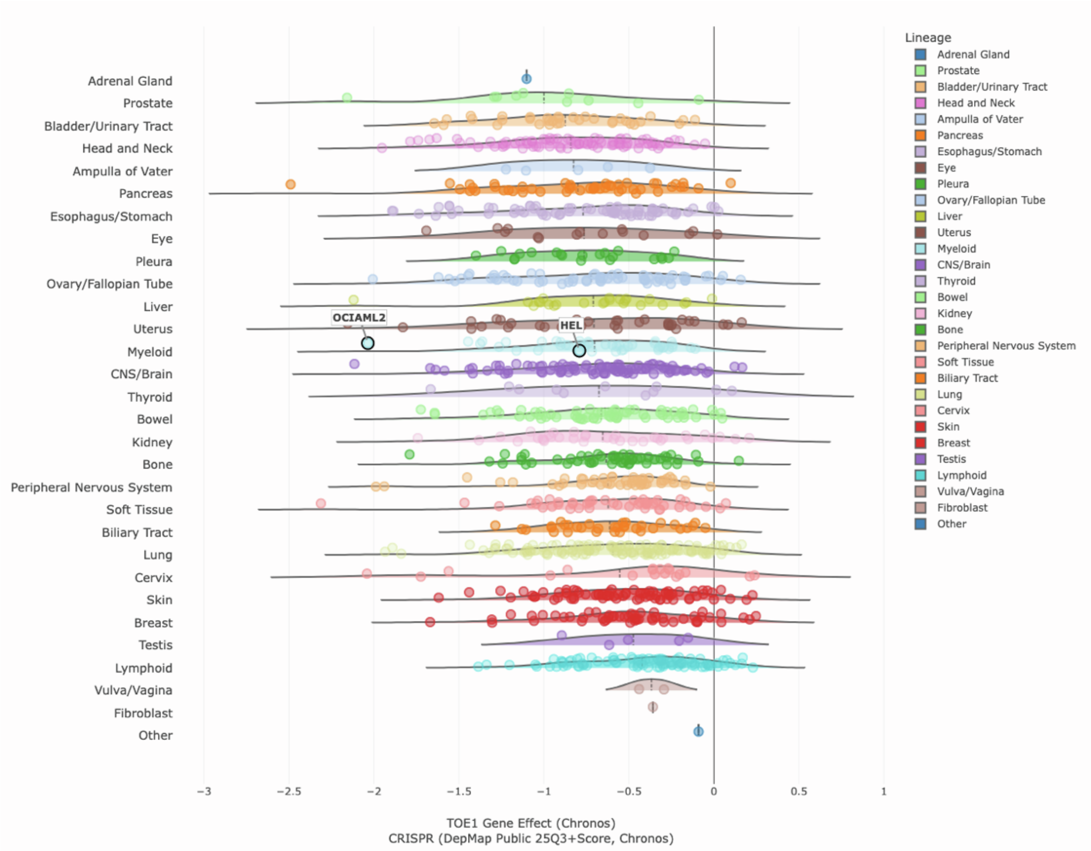

Supplemental Figure S3. Dependency profiles of TOE1 across a panel of cancer cell lines. Box and whisker plots representing Chronos dependency scores (based on cell depletion assays) of cancer cell lines derived from the DepMap CRISPR 23Q2+ data set(Institute, 2021). Numbers of cell lines within each lineage are shown in brackets. A Chronos score of -1 depicts the median of all pan-essential genes (represented as a red line). Lower Chronos scores represent a higher likelihood of *TOE1* dependency demonstrated by the cell line. OCI-AML2 and HEL positions are marked with Gene Effect (Chronos) scores of -2.01 and -0.791, respectively.

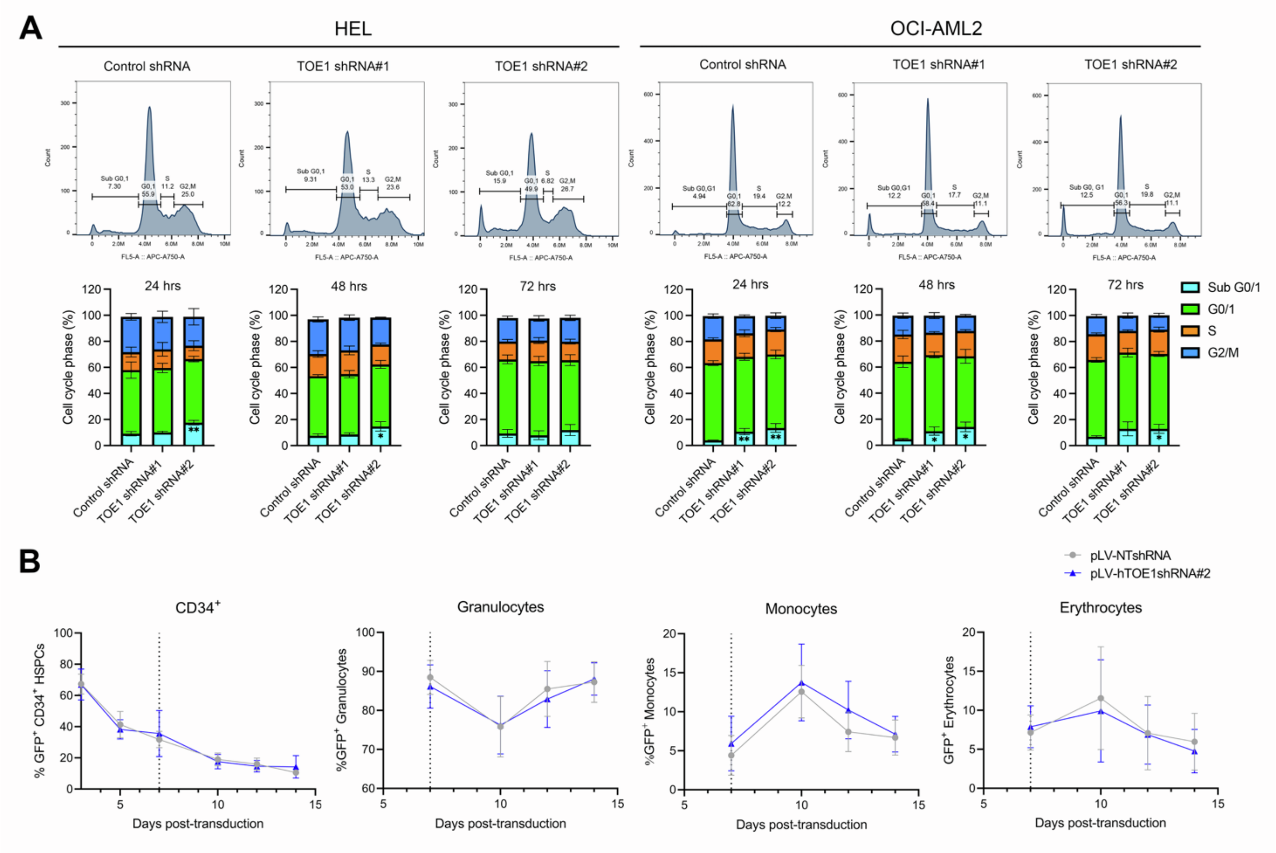

Supplemental Figure S4. TOE1 does not impact cell cycle status or myeloid differentiation in haematopoietic cells. (A) Representative flow cytometric DNA histograms demonstrating cell cycle stages at 48 hours in culture and summary graphs depicting the proportion (%) of cells within each of the sub-G0/G1, G0/1, S and G2/M phases represented over a period of 72 hours in culture in HEL and OCI-AML2 cells (n=3). **(B)** Vertical line at day 7 post-transduction represents initiation of steady state differentiation in $CD34^+$ HSPCs. Lineage discrimination markers were utilised to quantify granulocytic, monocytic and erythrocytic populations utilising flow cytometry (n=3). Error bars indicate mean ± 1SD. Statistical significance is denoted as *p<0.05, **p<0.01 (Student's *t*-test).

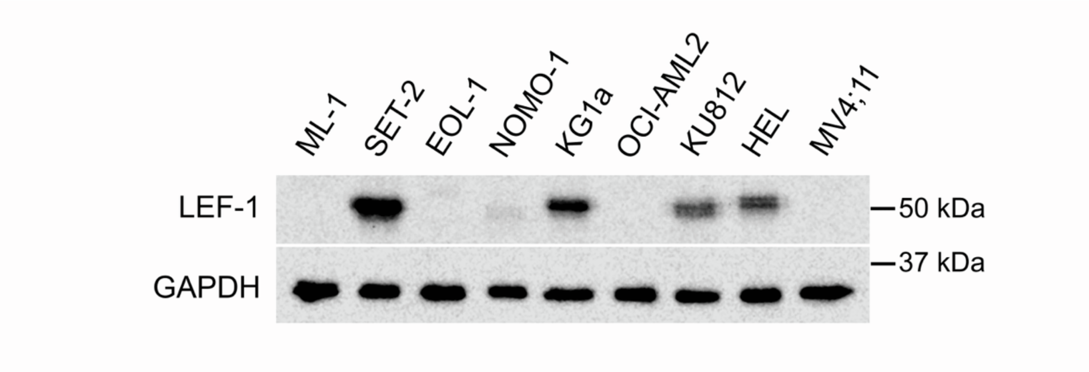

Supplemental Figure S5. Immunoblot demonstrating the protein expression of LEF-1 across a panel of myeloid cell lines. GAPDH was utilised as the loading control.

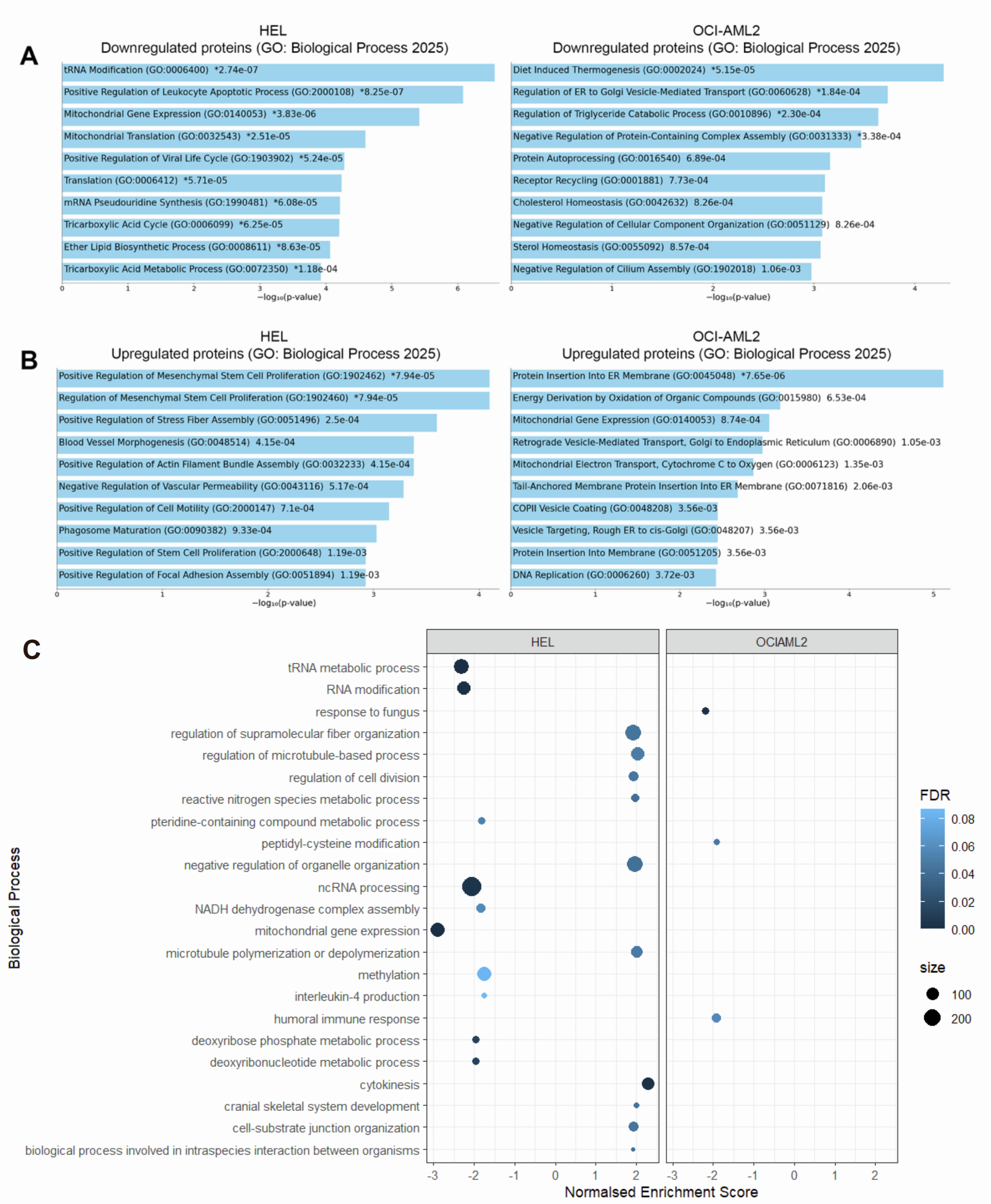

Supplemental Figure S6. Biological Process annotations of enriched proteins in mass spectrometry analysis. Gene ontology overrepresented terms associated with significantly **(A)** downregulated proteins and significantly **(B)** upregulated proteins in response to TOE1 depletion are shown. Statistical significance, defined by adjusted p-values <0.05 derived from Benjamini-Hochberg procedure, are highlighted with an asterix (*). **(C)**

TOE1 regulates the proliferation of hematopoietic cells
Park H, Sevim O et al, Stem Cell Reports, 2026
Supplementary information
Gene set enrichment analysis of HEL and OCI-AML2 cell lines showing 'Biological Processes' significantly altered by TOE1 depletion.

Supplemental Table S6. Common significantly downregulated peptides associated with TOE1 knockdown in myeloid cells.

Accession number	Protein name	Gene name	HEL abundance ratio	OCI-AML2 abundance ratio	HEL Adjusted P-value	OCI-AML2 Adjusted P-value
Q5EBM0	UMP-CMP kinase 2, mitochondrial	CMPK2	0.545	0.774	0.00298543	0.04657373
Q14764	Major vault protein	MVP	0.55	0.866	0.02459156	0.04157605
Q14651	Plastin-1	PLS-1	0.572	0.532	0.00664552	0.04445875
Q96PZ0	Pseudouridylate synthase 7 homolog	PUS-7	0.619	0.717	0.00160676	0.02206208
Q13177	Serine/threonine-protein kinase PAK 2	PAK2	0.629	0.615	0.01342231	0.02284624
C9JG97	Angio associated migratory cell protein	AAMP	0.703	0.949	0.04527474	0.00841372
D6RHI9	Ribonuclease T2	RNASE T2	0.765	0.826	0.0289509	0.04961872
A0A994J7E5	Centrosomal protein of 97 kDa	CEP97	0.796	0.782	0.04982668	0.00811732
P53602	Diphosphomevalonate decarboxylase	MVD	0.84	0.862	0.00228305	0.0207019

Supplementary Table S7. Common significantly upregulated peptides associated with TOE1 knockdown in myeloid cells.

Accession number	Protein name	Gene name	HEL abundance ratio	OCI-AML2 abundance ratio	HEL Adjusted P-value	OCI-AML2 Adjusted P-value
Q8WVM8	Sec1 family domain-containing protein 1	SCFD1	1.087	1.324	0.0359945	0.04336524
P50416	Carnitine O-palmitoyltransferase 1, liver isoform	CPT1A	1.137	1.294	0.0025737	0.00805737
Q969V3	BOS complex subunit NCLN	NCLN	1.161	1.229	0.024636	0.01773498
Q8IWB9	Testis-expressed protein 2	TEX2	1.388	1.123	0.01967537	0.03053523
Q96ME1	F-box/LRR-repeat protein 18	FBXL18	1.392	1.133	0.01684191	0.04806705
Q71UI9	Histone H2A.V	H2AZ2	1.537	1.774	0.03259417	0.03961278
E9PFN4	Anion exchange protein	SLC4A7	1.832	1.462	0.03771812	0.03188565
Q99523	Sortilin	SORT1	2.007	1.567	0.00590551	0.04473766

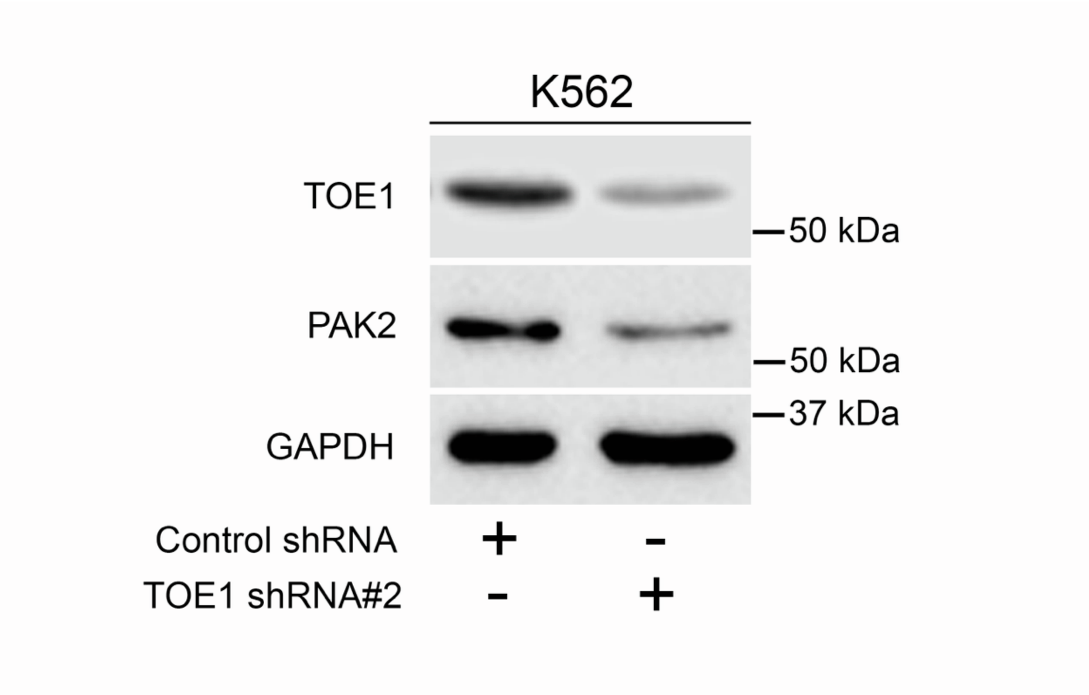

Supplemental Figure S7. Immunoblot demonstrating the protein expression of PAK2 in TOE1 depleted K562 cells. GAPDH was utilised as the loading control.

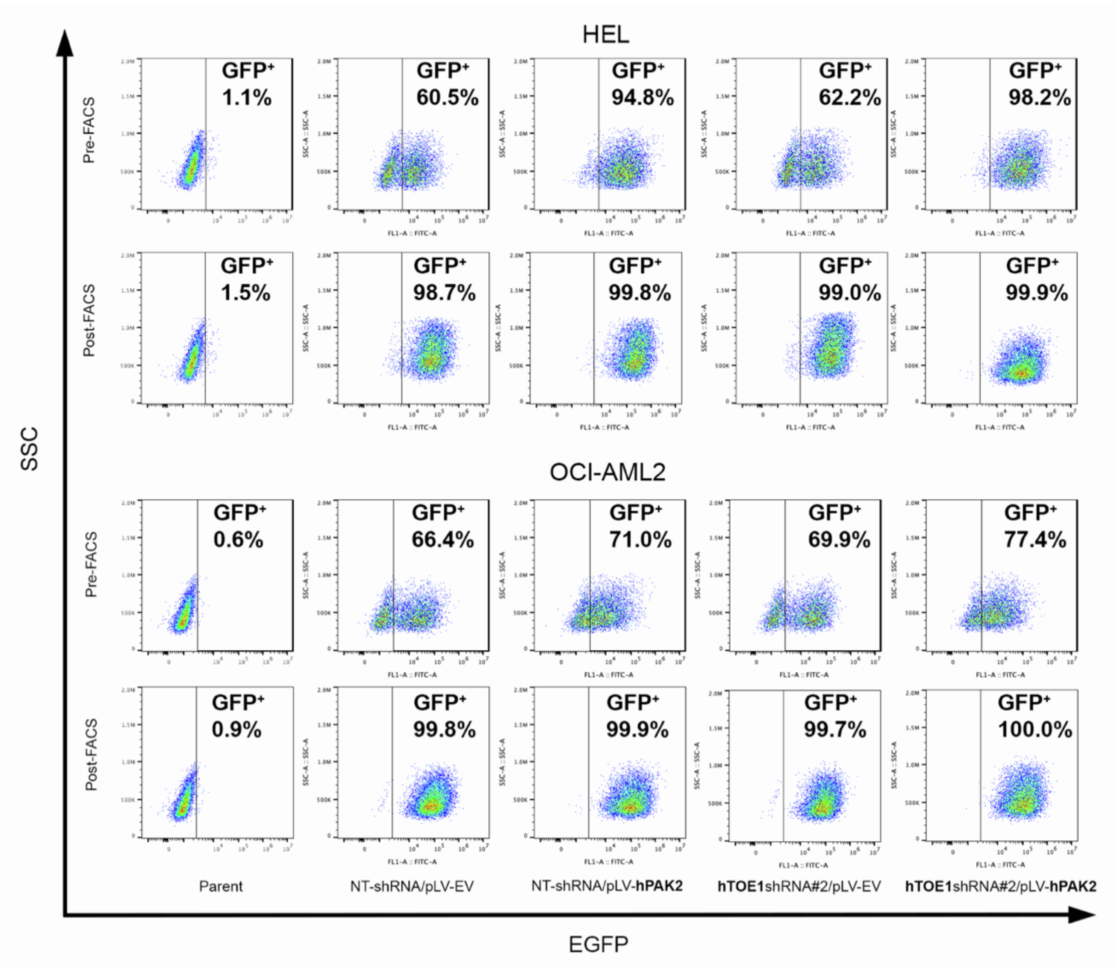

Supplemental Figure S8. Assessment of GFP$^+$ in lentivirally transduced myeloid leukaemia cells. Flow cytometric plots showing the proportion of enhanced green fluorescent protein (EGFP) cells pre- and post-FACS sorting as an assessment of lentiviral transduction efficiency. The negative EGFP threshold was determined utilising untransduced HEL and OCI-AML2 cells.

TOE1 regulates the proliferation of hematopoietic cells
Park H, Sevim O et al, Stem Cell Reports, 2026
Supplementary information

Supplementary methods

Primary samples

Mononuclear cells (MNCs) with viability >80% following isolation via density gradient separation using Ficoll-Hypaque (Merck-Millipore, Gillingham, Dorset) were included in the study and cryopreserved in liquid nitrogen until experimental use. The CD34^{+} HSPC fraction was enriched to >80% purity as previously described(Morgan et al., 2013), from cryopreserved cord blood MNC preparations using MiniMACS CD34 microbeads (Miltenyi Biotec, Woking, Surrey) according to the manufacturer's instructions and confirmed by flow cytometric assessment of CD34 positivity.

Cell culture and drug treatments

The myeloid cell lines K562, HL60, HEL, U937, PLB-985, NOMO1, OCI-AML3, EOL-1, ML-1, THP-1, KU812 (The European Collection of Authenticated Cell Cultures) and OCI-AML2, MV4;11, KG1, KG1a SET-2, NB4 and Mono-Mac-6 (Leibniz Institute DSMZ-German Collection of Microorganisms and Cell Cultures GmbH) were confirmed mycoplasma-free (Lonza, Basel, Switzerland) and authenticated via the Eurofins short-tandem repeat (STR) analytical service prior to project start. All cell lines were cultured in sterile non-coated tissue culture flasks (Sarstedt) with Roswell Park Memorial Institute-1640 media (RPMI; Merck-Millipore, Dorset, UK), supplemented with 2mM L-glutamine (Merck-Millipore) and 100IU/mL penicillin/100µg/mL streptomycin (Merck-Millipore) and maintained at 37°C under a humidified atmosphere of 5% carbon dioxide (CO_2). FBS (Biosera, Cholet, France) was supplemented at 20% for the culture of KG1, KG1a, and SET-2 cell lines, whilst all other cultures were supplemented at 10%. K562 and HEL cells harbouring the β-catenin-activated reporter (BAR) or mutant 'found unresponsive' control (fuBAR) were generated previously (Morgan et al., 2019). All cultures were

maintained at a density between 1-10x10^5/mL and all experiments datasets conducted within 15 passages from cryopreservation removal to limit genetic variation. Purified human CB CD34$^+$ HSPCs were maintained as previously(Wagstaff et al., 2025), at 5x10^5/ml in StemSpan SFEMII (StemCell Technologies, Cambridge, Cambridgeshire) supplemented with human recombinant 150ng/ml FLT3L, 150ng/ml SCF, and 20ng/ml TPO (Proteintech, Manchester, UK) following isolation into *in vitro* liquid culture.

TMT Labelling and High pH reversed-phase chromatography

Aliquots of 50µg of each sample were digested with trypsin (1.25µg trypsin; 37°C, overnight), labelled with Tandem Mass Tag (TMT) six plex reagents according to the manufacturer's protocol (Thermo Fisher Scientific) and the labelled samples pooled. The pooled sample was desalted using a SepPak cartridge according to the manufacturer's instructions (Waters, Milford, Massachusetts, USA). Eluate from the SepPak cartridge was evaporated to dryness and resuspended in buffer A (20 mM ammonium hydroxide, pH 10) prior to fractionation by high pH reversed-phase chromatography using an Ultimate 3000 liquid chromatography system (Thermo Fisher Scientific). In brief, the sample was loaded onto an XBridge BEH C18 Column (130Å, 3.5 µm, 2.1 mm X 150 mm, Waters, UK) in buffer A and peptides eluted with an increasing gradient of buffer B (20 mM Ammonium Hydroxide in acetonitrile, pH 10) from 0-95% over 60 minutes. The resulting fractions (concatenated into 15 in total) were evaporated to dryness and resuspended in 1% formic acid prior to analysis by nano-LC MSMS using an Orbitrap Fusion Lumos mass spectrometer (Thermo Scientific).

Nano-LC Mass Spectrometry

High pH RP fractions were further fractionated using an Ultimate 3000 nano-LC system in line with an Orbitrap Fusion Lumos mass spectrometer (Thermo Scientific). In brief, peptides in 1% (vol/vol) formic acid were injected onto an

Acclaim PepMap C18 nano-trap column (Thermo Scientific). After washing with 0.5% (vol/vol) acetonitrile 0.1% (vol/vol) formic acid peptides were resolved on a 500 mm × 75 μm Acclaim PepMap C18 reverse phase analytical column (Thermo Scientific) over a 150 min organic gradient, using 7 gradient segments (1-6% solvent B over 1min., 6-15% B over 58min., 15-32%B over 58min., 32-40%B over 5min., 40-90%B over 1min., held at 90%B for 6min and then reduced to 1%B over 1min.) with a flow rate of 300 nl min^{-1}. Solvent A was 0.1% formic acid and Solvent B was aqueous 80% acetonitrile in 0.1% formic acid. Peptides were ionized by nano-electrospray ionization at 2.0kV using a stainless-steel emitter with an internal diameter of 30 μm (Thermo Scientific) and a capillary temperature of 300°C. All spectra were acquired using an Orbitrap Fusion Lumos mass spectrometer controlled by Xcalibur 3.0 software (Thermo Scientific) and operated in data-dependent acquisition mode using an SPS-MS3 workflow. FTMS1 spectra were collected at a resolution of 120 000, with an automatic gain control (AGC) target of 400 000 and a max injection time of 100ms. Precursors were filtered with an intensity threshold of 5000, according to charge state (to include charge states 2-7) and with monoisotopic peak determination set to Peptide. Previously interrogated precursors were excluded using a dynamic window (60s +/-10ppm). The MS2 precursors were isolated with a quadrupole isolation window of 0.7m/z. ITMS2 spectra were collected with an AGC target of 10 000, max injection time of 70ms and CID collision energy of 35%. For FTMS3 analysis, the Orbitrap was operated at 30 000 resolution with an AGC target of 50 000 and a max injection time of 105ms. Precursors were fragmented by high energy collision dissociation (HCD) at a normalised collision energy of 60% to ensure maximal TMT reporter ion yield. Synchronous Precursor Selection (SPS) was enabled to include up to 10 MS2 fragment ions in the FTMS3 scan. The mass spectrometry proteomics data have been deposited to the ProteomeXchange Consortium via the PRIDE partner(Perez-Riverol et al., 2025) repository with the dataset identifier PXD070891.

Data Analysis

The raw data files were processed and quantified using Proteome Discoverer software v2.4 (Thermo Scientific) and searched against the UniProt Human database (downloaded January 2025: 83095 entries) using the SEQUEST HT algorithm. Peptide precursor mass tolerance was set at 10ppm, and MS/MS tolerance was set at 0.6Da. Search criteria included oxidation of methionine (+15.995Da), acetylation of the protein N-terminus (+42.011Da), methionine loss from the protein N-terminus (-131.04Da) and methionine loss plus acetylation of the protein N-terminus (-89.03Da) as variable modifications and carbamidomethylation of cysteine (+57.021Da) and the addition of the TMT mass tag (+229.163Da) to peptide N-termini and lysine as fixed modifications. Searches were performed with full tryptic digestion and a maximum of 2 missed cleavages were allowed. The reverse database search option was enabled, and all data was filtered to satisfy false discovery rate (FDR) of 5%.

Lentivirus generation and transduction

Briefly, 25cm^2 TC-treated flasks (Sarstedt) were poly-L-lysine-coated (Merck-Millipore) and seeded with 4x10^6 HEK293T packaging cells overnight targeting 80-90% confluence the following day. HEK293T cells were transfected with 4µg psPAX2 and 2.2µg pSL3 (pMD.2G) lentiviral packaging plasmids (Addgene), along with 2.1µg of the desired transfer vector (**Supplemental Table S4**) using LipofectamineTM 3000 transfection reagent (Thermo Fisher Scientific). Lentiviral harvests were collected at 24- and 48-hours post-transfection and snap frozen in liquid nitrogen followed by storage at -80°C. For myeloid cell line or CD34$^+$ HSPC transduction, target lentiviruses were sedimented to the base of 24-well tissue culture dishes (Thermo Fisher Scientific) coated with 40µg/ml retronectin (Takara Bio, London, UK) at 2,000g for 90 minutes, and 1-5x10^5 cells in log phase growth incubated with viral or

non-viral (selection controls) containing wells overnight. Transduced cells were selected through 1μg/mL puromycin (Merck-Millipore) treatment, and/or GFP enrichment via fluorescence activated cell sorting (FACS), depending on target vector. Upon selection completion (as deduced from non-transduced matched lines), target gene modulation was assessed via immunoblotting or flow cytometric assessment of Green Fluorescent Protein (GFP) positivity.

References

Evans, R., O'Neill, M., Pritzel, A., Antropova, N., Senior, A., Green, T., Žídek, A., Bates, R., Blackwell, S., Yim, J., et al. (2022). Protein complex prediction with AlphaFold-Multimer. bioRxiv, 2021.2010.2004.463034. 10.1101/2021.10.04.463034.

Institute, B. (2021). DepMap Portal https://depmap.org/portal/gene/TOE1?tab=overview.

Jumper, J., Evans, R., Pritzel, A., Green, T., Figurnov, M., Ronneberger, O., Tunyasuvunakool, K., Bates, R., Žídek, A., Potapenko, A., et al. (2021). Highly accurate protein structure prediction with AlphaFold. Nature *596*, 583-589. 10.1038/s41586-021-03819-2.

Krissinel, E., and Henrick, K. (2007). Inference of macromolecular assemblies from crystalline state. J Mol Biol *372*, 774-797. 10.1016/j.jmb.2007.05.022.

Morgan, R.G., Liddiard, K., Pearn, L., Pumford, S.L., Burnett, A.K., Darley, R.L., and Tonks, A. (2013). gamma-Catenin is expressed throughout normal human hematopoietic development and is required for normal PU.1-dependent monocyte differentiation. Leukemia *27*, 2096-2100. 10.1038/leu.2013.96.

Morgan, R.G., Ridsdale, J., Payne, M., Heesom, K.J., Wilson, M.C., Davidson, A., Greenhough, A., Davies, S., Williams, A.C., Blair, A., et al. (2019). LEF-1 drives aberrant beta-catenin nuclear localization in myeloid leukemia cells. Haematologica *104*, 1365-1377. 10.3324/haematol.2018.202846.

Perez-Riverol, Y., Bandla, C., Kundu, D.J., Kamatchinathan, S., Bai, J., Hewapathirana, S., John, N.S., Prakash, A., Walzer, M., Wang, S., and Vizcaino, J.A. (2025). The PRIDE database at 20 years: 2025 update. Nucleic Acids Res *53*, D543-D553. 10.1093/nar/gkae1011.

TOE1 regulates the proliferation of hematopoietic cells
Park H, Sevim O et al, Stem Cell Reports, 2026
Supplementary information

Wagstaff, M., Sevim, O., Goff, A., Raynor, M., Park, H., Mancini, E.J., Nguyen, D.T.T., Chevassut, T., Blair, A., Castellano, L., et al. (2025). β-Catenin interacts with canonical RBPs including MSI2 to associate with a Wnt signalling mRNA network in myeloid leukaemia cells. Oncogene. 10.1038/s41388-025-03415-y.
