# Supplementary material for: TOE1 is a β-catenin interacting protein regulating the proliferation of hematopoietic cells through PAK2 modulation
Source: Stem Cell Reports. 2026 Apr 23;21(5):102894. doi: 10.1016/j.stemcr.2026.102894 (PMC13163219; doi:10.1016/j.stemcr.2026.102894)
Supplement: Document S2. Article plus supplemental information [file mmc4.pdf]

STEM CELL REPORTS
Article
OPEN ACCESS

TOE1 is a β-catenin interacting protein regulating the proliferation of hematopoietic cells through PAK2 modulation

Hyun Park,[1,2,3,4,9] Okan Sevim,[1,5,9] Megan Wagstaff,[1] Aaron Goff,[1] David A. Palmer,[2,3] Bomee Kim,[3] Kate Heesom,[6] Allison Blair,[7] Sarah F. Newbury,[2] Ethan L. Morgan,[1,8] Benjamin P. Towler,[1] Timothy J. Chevassut,[2,3,10] and Rhys G. Morgan[1,10,11,*]
[1]School of Life Sciences, University of Sussex, Brighton, UK
[2]Clinical and Experimental Medicine, Brighton & Sussex Medical School, Brighton, UK
[3]University Hospitals Sussex NHS Foundation Trust, Brighton, UK
[4]Medical Sciences Division, University of Oxford, Oxford, UK
[5]Faculty of Medicine, Pamukkale University, Denizli, Türkiye
[6]University of Bristol Proteomics Facility, Bristol, UK
[7]Bristol Institute for Transfusion Sciences, NHS Blood & Transplant, Bristol, UK
[8]Tumour Virology Group, The Cyprus Institute of Neurology & Genetics, Nicosia, Cyprus
[9]These authors contributed equally
[10]These authors contributed equally
[11]Lead contact
*Correspondence: rhys.morgan@sussex.ac.uk
https://doi.org/10.1016/j.stemcr.2026.102894

SUMMARY

Acute myeloid leukemia (AML) is an aggressive hematological malignancy frequently exhibiting deregulated expression/activity/localization of the Wnt signaling mediator β-catenin. To derive more effective β-catenin targeting strategies, we previously interrogated its interaction network in myeloid cells and identified several putative novel interacting partners, including Target of EGR1 (TOE1); a deadenylase with unknown function in hematological tissue. This study aimed to define TOE1 function in hematopoietic cells and uncover its molecular targets. TOE1 interacted with β-catenin in both primary and immortalized AML cells, and impacted Wnt signaling output through the modulation of lymphoid enhancer-binding factor-1 (LEF-1). AML samples exhibited deregulated TOE1 expression versus normal hematopoietic stem/progenitor cells (HSPCs), and TOE1 depletion suppressed the proliferation of myeloid leukemia cell lines, and primary human HSPCs, partly through a p21-activated-kinase 2 (PAK2) mediated mechanism. In summary, these data reveal TOE1 as a novel regulator of hematopoietic cell proliferation via the modulation of important growth-regulating pathways.

INTRODUCTION

Acute myeloid leukemia (AML) is an aggressive clonal disorder of hematopoietic stem/progenitor cells (HSPCs) resulting in arrested myeloid development and enhanced self-renewal properties. Despite the emergence of several new targeted therapies (Halik et al., 2022), the prognosis for most patients remains poor, and the heterogeneity of AML demands a larger range of targeted molecular therapies with broad applicability. Dysregulated signal transduction is a hallmark of AML biology and frequently the target of novel therapy design, as evidenced by novel agents targeting FLT3 and Hedgehog signaling (Dohner et al., 2021). Canonical Wnt/β-catenin signaling is important for the maintenance and development of HSPCs (Luis et al., 2012a, 2012b; Reya et al., 2003; Shooshtarizadeh et al., 2019; Willert et al., 2003), and is frequently deregulated in multiple AML subtypes (Wagstaff et al., 2022), where it sustains leukemia stem cell (LSC) activity (Dietrich et al., 2014; Wang et al., 2010; Yeung et al., 2010), and drug resistance (Fong et al., 2015; Perry et al., 2020). Efforts to pharmacologically target the central mediator β-catenin through its stability or transcriptional activity have shown promise to date (Wagstaff et al., 2022), but have been hampered by an incomplete understanding of β-catenin's context-specific interactions.

Our previous interrogation of the β-catenin interaction network in hematopoietic cells revealed new interaction partners such as WT1 (Wagstaff et al., 2023) and MSI2 (Wagstaff et al., 2025) as part of a dense network of RNA-binding proteins (RBPs) and subsequently enriched mRNA with β-catenin (Morgan et al., 2019; Wagstaff et al., 2025), suggestive of a novel post-transcriptional role in hematological cells (Sevim et al., 2025). One such novel partner identified was Target of EGR1 (TOE1), a 510-amino acid member of the Asp-Glu-Asp-Asp (DEDD) family of deadenylases with previously uncharacterized function in hematopoietic cells. The TOE1 gene was only identified and characterized relatively recently, being identified as a suppressor of cell growth mediated by EGR1 via the induction of the cell cycle regulator p21 (De Belle et al., 2003). Subsequent studies have more definitively characterized TOE1 as a Cajal body localized protein with 3′ exonuclease function controlling the maturation/stability of small nuclear RNAs (snRNAs) (Fong et al., 2013; Lardelli and Lykke-Andersen, 2020; Son et al., 2018; Wagner et al.,

© 2026 International Society for Stem Cell Research. Published by Elsevier Inc.
This is an open access article under the CC BY license (http://creativecommons.org/licenses/by/4.0/).

2007), and disruption of this function through biallelic loss-of-function *TOE1* mutations was identified as a fundamental driver of the neurodegenerative syndrome Pontocerebellar Hypoplasia type 7 (PCH7) (Lardelli et al., 2017; Ma et al., 2024; Wang et al., 2023). Additional studies have begun to unravel the complexity of TOE1 function with diverse roles (RNA-related or not) reported in telomere maintenance (Deng et al., 2019), p53 transcriptional activity (Sperandio et al., 2009), and inhibition of HIV transcription and replication in infected T-cells (Sperandio et al., 2015).

The role of TOE1 in human cancer or stem cell biology remains underexplored. Of the limited studies available, one report in gastric cancer indicates a tumor suppressor role for TOE1 (Sun et al., 2025), while in hepatocellular carcinoma (HCC) TOE1 may serve oncogenic roles (Jakobi et al., 2003), particularly through an MYC-STAMBPL1 axis promoting EGFR stability and subsequent Lenvatinib sensitivity (Zhang et al., 2024). Indeed, small molecule inhibitors for TOE1 have been screened and predicted to have anticancer activity (Kehinde et al., 2024). The aim of this study was to assess any molecular crosstalk between β-catenin and TOE1 in myeloid cells, and characterize the wider role of TOE1 in a hematopoietic setting for the first time.

RESULTS

TOE1 is predominantly nuclear localized in myeloid cells and interacts with cytosolic and nuclear β-catenin

Our previous β-catenin interactome data suggested TOE1 interactions across multiple myeloid cell lines, including K562, HEL, THP-1, and HL-60 (Figure 1A), but not SW620 colorectal cancer cells (Morgan et al., 2019), implying a potential tissue-dependent interaction. TOE1 also has a low presence on the CRAPome database (31/716 = <5%) (Mellacheruvu et al., 2013), reducing the probability it represents a background contaminant and rationalizing further investigation. To select models for onward study, we initially performed a screen of TOE1 expression across 18 myeloid cell lines and observed ubiquitous expression across all cell lines (with the exception of U937 and Mono-Mac-6) alongside its functional homolog PARN, and a high degree of co-expression with β-catenin (Figure 1B). To confirm interaction with β-catenin we performed reciprocal TOE1 Co-IPs in K562 and HEL cells and observed consistent enrichment of β-catenin under both basal and Wnt signaling-stimulated conditions (Figure 1C). Since TOE1 has not been studied in a hematopoietic context, we sought to characterize its subcellular localization. Through nuclear/cytosolic fractionation assays, we observed TOE1 to be a predominantly nuclear-localized protein (apart from HL-60) and its localization was unaffected by β-catenin stabilization via GSK3β inhibition (Figure 1D). Confocal microscopy confirmed predominant nuclear TOE1 localization with some intense nucleolar-like foci consistent with its previously reported inclusion into Cajal bodies (Figure 1E) (Fong et al., 2013). β-Catenin levels were elevated in the nucleus upon Wnt signaling activation (Figure 1F), and compartment-specific TOE1 Co-IP confirmed interaction with β-catenin in both the cytoplasm and nucleus of myeloid cells (Figure 1G). Finally, given β-catenin and TOE1's previous associations with mRNA (Deng et al., 2019; Kim et al., 2012; Lee and Jeong, 2006; Ma et al., 2024; Son et al., 2018), we ascertained whether the interaction was a consequence of mRNA co-occupancy. Despite complete digestion of RNA in Co-IP input samples via RNaseA treatment (Figure 1H), the β-catenin:TOE1 interaction remained in both K562 and HEL cells (Figure 1I). Furthermore, AlphaFold modeling also predicted against a direct protein interaction between β-catenin and TOE1 (Figure S1A), suggesting these proteins could be part of a multi-protein complex. Taken together, these data confirm that β-catenin forms an RNA-independent indirect interaction with TOE1 in myeloid cells.

TOE1 is overexpressed in AML and associated with poor risk and lower overall survival

Following the confirmation of TOE1's interaction with β-catenin in myeloid cells, we next explored the clinical relevance of TOE1 in AML. Using the adult AML TCGA New England Journal of Medicine (NEJM) 2013 dataset from CBioPortal (Cerami et al., 2012), we found that higher *TOE1* mRNA expression was associated with poor risk AML (Figure 2A) and lower overall survival (Figure 2B), likely driven by the significant enrichment of *TP53* mutations (Figure S2; Table S5), a dismal prognostic marker in AML (Welch, 2018). We next examined the protein expression of TOE1 and β-catenin using a previously interrogated panel of primary AML patient samples (Wagstaff et al., 2023). We observed faint but detectable levels of TOE1 in normal cord blood (CB)-derived $CD34^+$ HSPC; however, TOE1 expression levels were higher in 25/28 (89.3%) of AML samples, often with multiple banding (Figure 2C). Across the panel, 19/28 (67.9%) samples exhibited co-expression of TOE1 and β-catenin, but there was no correlation in the level of the two proteins ($R = 0.06$, $p = 0.787$). Finally, in a primary AML patient sample expressing abundant levels of both β-catenin and TOE1, where ample cellular material existed (patient #20; MLL rearrangement t(9;11) with M5a morphology), we evaluated the β-catenin:TOE1 interaction through TOE1 Co-IP, and observed substantial enrichment of β-catenin (Figure 2D). In summary, these data indicate that TOE1 is dysregulated in AML with the potential to impact survival and is worthy of further investigation.

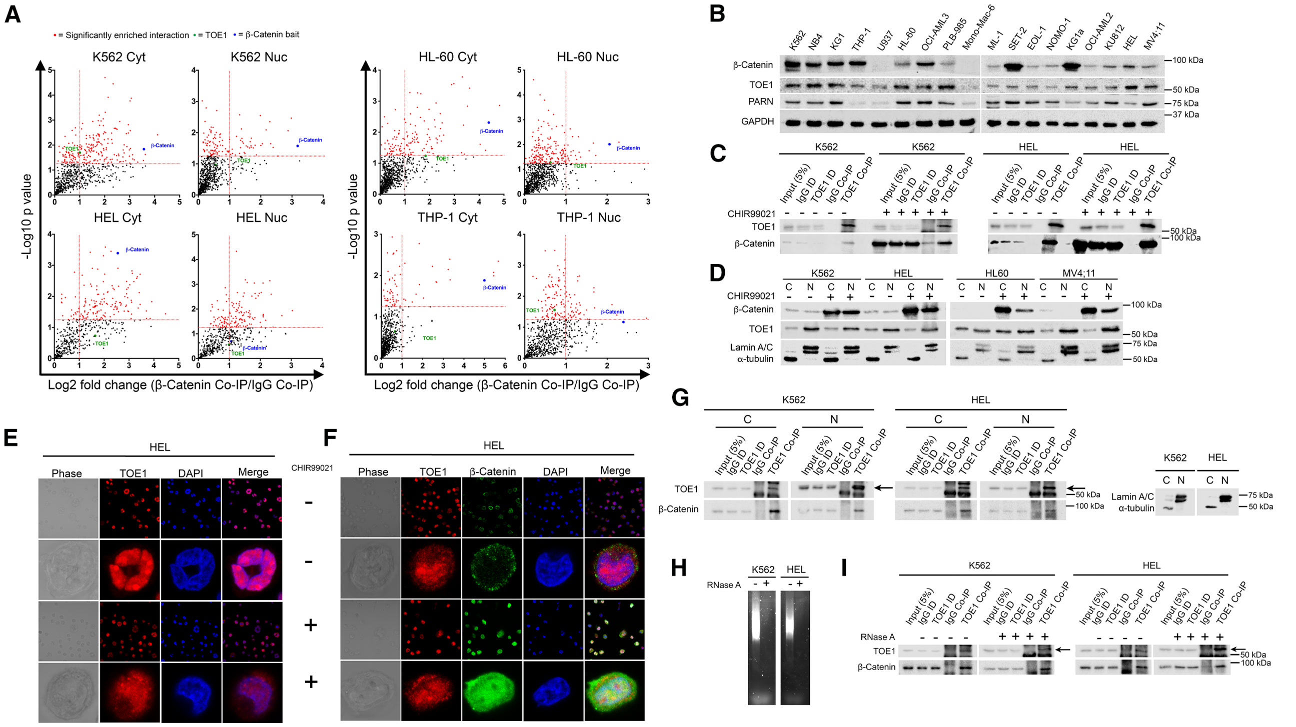

Figure 1. β-Catenin interacts with TOE1 in myeloid leukemia cells

(A) Scatterplots demonstrate β-catenin protein interactions detected in K562 cytosolic/nuclear, HEL cytosolic/nuclear, HL-60 cytosolic/nuclear, and THP-1 cytosolic/nuclear fractions. The vertical dashed red line indicates the threshold for 2-fold change in protein binding at log2 (=1) in β-catenin CoIP relative to IgG CoIP, $n = 3$. The horizontal red line represents the threshold for significant interactions at $p = 0.05$ on $\log_{10}$ scale (=1.3). Highlighted red dots indicate statistically significant interactions, with TOE1 labeled in green. The remaining black dots represent other proteins detected in the mass spectrometry analysis. Fold change values less than 0 are not shown as these represent isotype Co-IP-enriched events.

(B) Immunoblot demonstrates the endogenous protein expression of β-catenin, TOE1, and PARN across a panel of 18 myeloid cell lines. GAPDH was utilized as the loading control. (C) Representative immunoblot shows the level of β-catenin protein present in TOE1 Co-IP derived from K562 and HEL cells under basal (DMSO) versus induced (5 μM CHIR99021 overnight) Wnt signaling conditions, ID = immunodepleted.

(D) Representative immunoblot shows the protein levels of β-catenin and TOE1 under basal (DMSO) versus induced (5 μM CHIR99021) Wnt signaling conditions in K562, HEL, HL-60 and MV4;11 cells. Lamin A/C and α-tubulin were utilized to indicate the loading and purity of the nuclear (N) and cytosolic (C) fractions respectively. Confocal microscopy laser scanning sections demonstrate the sub-cellular localization of (E) TOE1 and (F) TOE1 in conjunction with β-catenin under basal (DMSO) versus induced (5 μM CHIR99021 overnight) Wnt signaling conditions in HEL cells. Phase (gray), TOE1 (red), β-catenin (green), DAPI (blue) and merged TOE1/DAPI images depicted.

(G) Representative immunoblot demonstrates the level of β-catenin present in TOE1 Co-IP derived from the cytosolic (C) and nuclear (N) fractions of K562 and HEL cells. Lamin A/C and α-tubulin were utilized to indicate the loading and purity of the N and C fractions respectively. Non-specific bands above and below the TOE1 band (57 kDa) were observed in the Co-IP analysis, with the specific TOE1 band represented by an arrow.

(H) Representative agarose gel electrophoresis images demonstrate RNA in K562/HEL whole-cell lysates treated with ±20 μg/mL RNase A overnight prior to TOE1 Co-IP analyses.

(I) Representative immunoblot shows the level of β-catenin protein present in TOE1 Co-IP derived from K562 and HEL cells ±20 μg/mL RNase A. Non-specific bands above and below the TOE1 band (57 kDa) were observed in the Co-IP analysis, with the specific TOE1 band represented by an arrow.

TOE1 regulates Wnt/β-catenin signaling through LEF-1 modulation

Following the confirmation of β-catenin:TOE1 interaction in clinical samples, we next explored the molecular consequence of this relationship through its impact on canonical Wnt signaling activity. To evaluate the role of TOE1 on Wnt signaling output, we generated TOE1 knockdown models in HEL and K562 cells using shRNAs and confirmed the reduction of TOE1 protein by immunoblotting, which was superior through shRNA#2 (Figure 3A). Using the

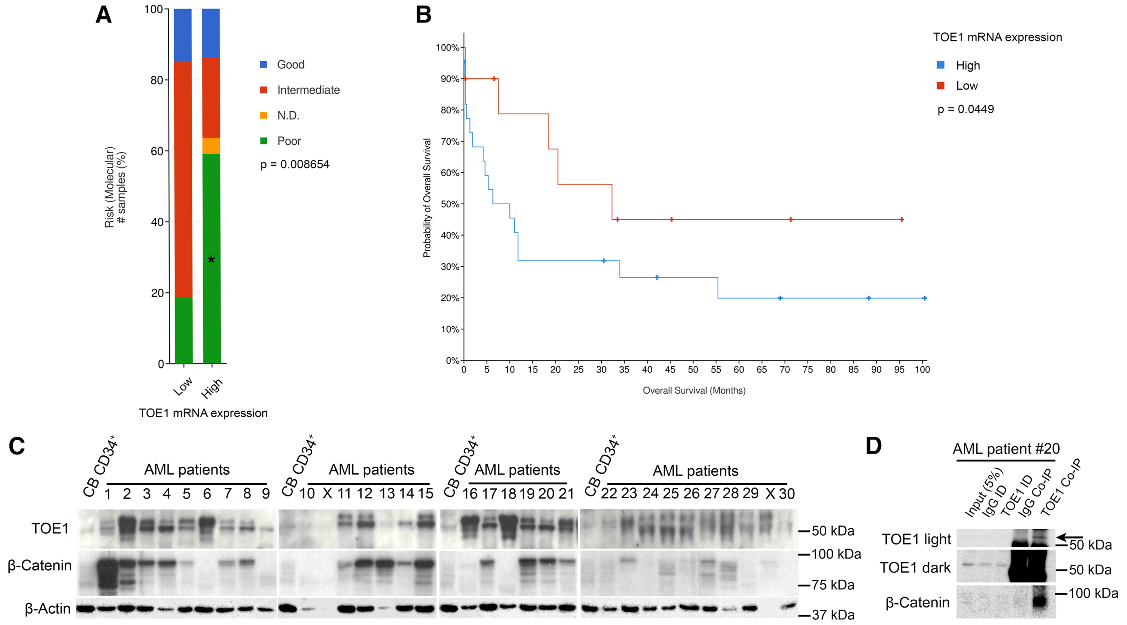

Figure 2. TOE1 is overexpressed in AML and associated with poor risk and lower overall survival

(A) Bar chart demonstrates the clinical characteristics of patients with AML based on the level of TOE1 mRNA expression.

(B) Kaplan-Meier curve shows the estimated probability of overall survival in patients with AML. *TOE1* mRNA levels were derived from the New England Journal of Medicine (NEJM) 2013 The Cancer Genome Atlas (TCGA) whole-exome sequencing dataset (Cerami et al., 2012; de Bruijn et al., 2023; Gao et al., 2013). Low *TOE1* mRNA derived via Z score <1; $n = 27$ versus high Z score ≥ 1; $n = 22$. PB, peripheral blood; WBC, white blood cell; N.D, not determined.

(C) Immunoblot shows the relative levels of β-catenin and TOE1 protein levels in 30 primary AML patient samples alongside cord blood-derived CD34^{+} HSPCs pooled from five independent cord blood samples. X = void samples due to insufficient β-actin levels, which were utilized as a loading control.

(D) Representative immunoblot shows the level of β-catenin protein present in TOE1 Co-IP derived from AML patient #20 (light and dark exposures shown). Non-specific bands above and below the TOE1 band (57 kDa) were observed in the Co-IP analysis, with the specific TOE1 band represented by an arrow.

β-catenin-activated reporter (BAR) adopted previously (Morgan et al., 2013, 2019; Wagstaff et al., 2023, 2025), we first assessed the impact of TOE1 depletion on Wnt signaling output (TCF/LEF activity) and observed a significantly attenuated capacity for Wnt signaling induction in HEL cells in response to the GSK3β inhibitor (and Wnt agonist) CHIR99021 (Figure 3B). To understand the underlying cause for this diminished Wnt signaling output, we examined the whole-cell expression of key Wnt signaling components. We observed no overall change in β-catenin level; however, we did observe a significant depletion in the level of the Wnt effector LEF-1, which correlated with the efficiency of TOE1 knockdown (Figure 3C).

Given TOE1's extensive characterization as an RNA interacting/modulating protein (Deng et al., 2019; Lardelli et al., 2017; Ma et al., 2024; Son et al., 2018), we next sought to ascertain the level of LEF-1 regulation by TOE1 modulation through the examination of *LEF1* transcript levels. In both K562 and HEL cells, we observed no change in *LEF1* levels in cells harboring TOE1 shRNA versus non-targeting (NT) shRNA controls (Figure 3D). In support of this finding, we also observed no strong association of TOE1 with RNA through both RNA immunoprecipitation (RIP) and crosslinking immunoprecipitation (CLIP) approaches (Figure S1B), in contrast to an RBP LIN28B RIP/CLIP, which pulled down abundant RNAs (Figure S1C). In the absence of *LEF1* mRNA regulation upon TOE1 depletion, we instead focused on protein regulation, given TOE1's recent association with EGFR protein stability (Zhang et al., 2024). Our previous data suggest LEF-1 has a very stable half-life (Wagstaff et al., 2025), and have further since confirmed that LEF-1 is not strongly lysosomally regulated (Figure S1D). However, we have shown that LEF-1 protein stability is modestly controlled by the proteasome in HEL and K562 following 8h MG132 exposure, in keeping with previous reports (Figure S1E) (Shao et al., 2021). To next ascertain a suitable window for assessing TOE1 impact on LEF-1 peptide stability, we performed a cycloheximide (CHX) chase assay and observed marked LEF-1 degradation following 8 h of translation inhibition through CHX

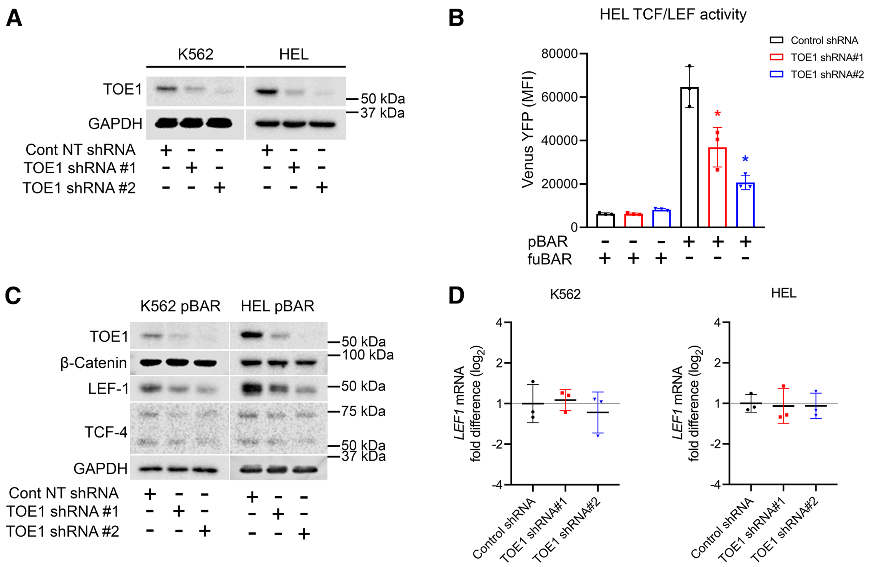

Figure 3. TOE1 regulates Wnt/β-catenin signaling through LEF-1 modulation

(A) Immunoblot demonstrates the levels of TOE1 expression in and K562 and HEL cells ± TOE1 shRNA. GAPDH was utilized as the loading control.

(B) Bar graph demonstrates the Venus YFP MFI from the BAR system in HEL cell lines + 5 μM CHIR99021 overnight upon TOE1 knockdown. Error bars indicate mean ± 1SD. Statistical significance is denoted as $*p < 0.05$, $n = 3$.

(C) Representative immunoblots demonstrate the whole-cell protein levels of key effector molecules implicated in Wnt signaling ± 5 μM DMSO/CHIR99021 in K562 cells and HEL cells ± TOE1 shRNA harboring the BAR reporter system. GAPDH was utilized as the loading control.

(D) Summary plot shows the fold change in *LEF1* mRNA expression in K562 and HEL cells ± TOE1 shRNA ($n = 3$). GAPDH was utilized as the reference gene.

(Figure 4A). Interestingly, TOE1 remained remarkably stable in both K562 and HEL cells even up to a maximum of 24 h of CHX exposure, suggesting TOE1 may serve important housekeeping functions in leukemia cells. We next examined the capacity of TOE1 to impact LEF-1 protein stability in HEL cells and observed that TOE1 depletion significantly reduced LEF-1 protein stability at 8 h CHX treatment (Figures 4B and 4C). In summary, these data together demonstrate that TOE1 may regulate Wnt/β-catenin signaling levels through LEF-1 protein stability/translation.

TOE1 regulates the growth and survival of hematopoietic cells

Having demonstrated that TOE1 is a β-catenin-interacting protein capable of influencing Wnt signaling output and is dysregulated in AML, we next sought to understand its independent functional roles in AML. Initial exploration into TOE1 function using the DepMap Cancer Dependency Map revealed that many cancer cell lines, including myeloid cells, exhibit TOE1 dependency with the largest perturbation effects observed with CRISPR/Cas9 modulation versus RNAi, presumably because of superior target depletion (Figure S3). Two of the AML cell lines, differing in origin, but predicted to be sensitive to TOE1 depletion, were OCI-AML2 and HEL; therefore, we introduced TOE1 shRNA into these lines (Figure 5A), and examined their growth over 72 h. We observed attenuated cell growth rates at 72 h in both cell lines harboring TOE1 shRNA versus NT shRNA controls, with the greatest perturbation occurring with the more efficient TOE1 shRNA#2 (Figures 5B and 5C). To examine the cause of depleted cell number, we examined apoptosis rates using Annexin V staining and observed significantly higher rates of early and late apoptosis across 72 h with TOE1 shRNA#2 in HEL cells, and significantly higher rates of late apoptosis with both TOE1 shRNAs in OCI-AML2, compared with NT shRNA controls (Figures 5D and 5E). Examination of cell cycle status through DRAQ5 staining revealed no substantial differences between TOE1 versus NT shRNA cells, although larger sub-G0 peaks were observed, consistent with a higher rate of apoptosis in TOE1-depleted HEL/OCI-AML2 cells (Figure S4A).

Finally, to see if the TOE1 regulation of cell growth or survival extended to normal healthy cells, we lentivirally transduced normal human CB-derived $CD34^+$ HSPCs with the optimal TOE1 shRNA#2 containing a GFP selectable marker (Figure 5G) and examined both growth rates and broad lineage commitment markers. TOE1 depletion resulted in a gradual depletion in overall GFP^+ levels from 7 days *in vitro* liquid culture (Figure 5H), which resulted in a significant fold-reduction in GFP^+ levels upon 14-days in steady state differentiation culture (Figure 5I). We observed no overt impact of TOE1 on the CD34 positivity of these cultures or broad commitment to monocytic ($CD13^+CD36^+$), granulocytic ($CD13^{+/-}CD36^-$) or erythroid lineages ($CD13^-CD36^+$), indicating that TOE1's primary influence is over growth/survival, rather than differentiation, of HSPC (Figure S4B). Taken together, these data indicate TOE1 positively regulates the growth and survival of both immortalized AML cell lines and normal healthy human HSPC.

TOE1 depletion reduces PAK2 protein abundance

Our previous analyses showed that TOE1 could impact LEF-1 expression, and we previously reported that LEF-1

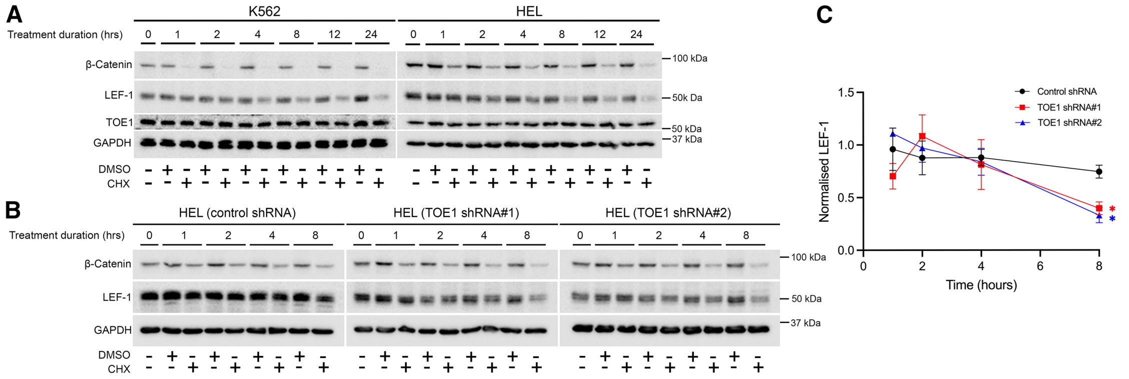

Figure 4. TOE1 regulates LEF-1 protein stability

(A) Representative immunoblots show the protein levels of LEF-1, β-catenin, and TOE1 in K562 and HEL cells ±100nM cycloheximide (CHX). GAPDH was utilized as the loading control.

(B) Representative immunoblots demonstrate the protein levels of LEF-1 and β-catenin in HEL cells ± TOE1 shRNA ±100nM CHX). GAPDH was utilized as the loading control.

(C) Line graph depicts LEF-1 levels in HEL cells ± TOE1 shRNA ± 100nM CHX calculated via densitometry analysis, normalized to the relative quantitation values of GAPDH at each time point and DMSO control values. Error bars indicate mean ± 1 standard error of the mean (SEM), with statistical significance denoted as $*p < 0.05$, $n = 3$.

regulates the proliferation of HEL cells (Morgan et al., 2019); however, we found that OCI-AML2 cells do not express LEF-1 (Figure S5). Therefore, to identify a more unifying mechanism mediating TOE1 control of hematopoietic cell proliferation/survival, we explored the proteome of TOE1-depleted cells using quantitative tandem-mass tag (TMT) labeled mass spectrometry (MS), given prior data showing TOE1 influences LEF-1 protein stability. We generated three experimental replicates for HEL and OCI-AML2 with confirmed TOE1 depletion prior to TMT-labeling and MS analysis (Figure 6A). MS data (deposited to the ProteomeXchange Consortium via the PRIDE partner repository with the dataset identifier PXD070891) revealed modest but significant alterations to protein abundance between control and TOE1 shRNA cells (Figures 6B and 6C), with 465 proteins downregulated, and 98 upregulated in HEL cells, and 117 downregulated, and 184 upregulated in OCI-AML2 cells (Figure 6C, Data S1). These proteins were associated with a diverse range of biological processes as revealed by Gene Ontology (GO) analysis including many RNA-related functions such as "*tRNA modification,*" "*translation,*"and "*mRNA Pseudouridine synthesis,*" as well as terms pertaining to proliferation including the "*regulation of mesenchymal stem cell proliferation*" and "*regulation of stem cell proliferation*" (Figure S6). Between both cell lines, there were a total of 9 commonly downregulated (Table S6) and 8 commonly upregulated proteins (Table S7) identified. Given the relatively small number of commonly regulated proteins identified from both cell lines, we took a targeted approach to the validation and functional interrogation of putative growth-regulating proteins. Given its known association with growth and survival (Jakobi et al., 2003; Rudel and Bokoch, 1997; Vilas et al., 2006), and the proliferation of murine HSPC (Zeng et al., 2015), we selected p21 (RAC1) activated kinase 2 (PAK2) for further investigation. Subsequent validation in HEL and OCI-AML2 via immunoblotting showed a modest but consistent decrease in PAK2 protein expression in response to TOE1 depletion (Figures 6D and 6E), with strong regulation also observed in K562 cells (Figure S7). In summary, these data show TOE1 depletion can modestly impact the cell proteome, including the regulation of putative growth/survival-regulating proteins such as PAK2.

PAK2 partially mediates the proliferative influence of TOE1 in normal hematopoietic and AML cells

Having shown that TOE1 depletion results in PAK2 reduction, we next interrogated whether this axis could regulate the growth/survival attenuation observed upon TOE1 loss. To address this, we first modulated PAK2 in both HEL and OCI-AML2 using prior-optimized shRNAs (Figure 7A) and assessed cell viability/growth. Despite presenting with the most efficient PAK2 depletion, shRNA#1 could not be progressed experimentally since selected cells grew too poorly. As observed in Figures 7B and 7C, PAK2 shRNAs #3 and #4 significantly curtailed cell growth rates in both cell lines but did not impact apoptosis. Since PAK2 appeared to be a positive regulator of proliferation, we next expressed ectopic PAK2 into HEL/OCI-AML2 cells harboring TOE1 shRNA#2 that were generated previously and examined whether proliferation could be recovered. Given TOE1 shRNA#2 cells were previously selected via puromycin treatment, ectopic PAK2 (prior optimized in Figure 7A) was delivered via a GFP selectable construct, and following

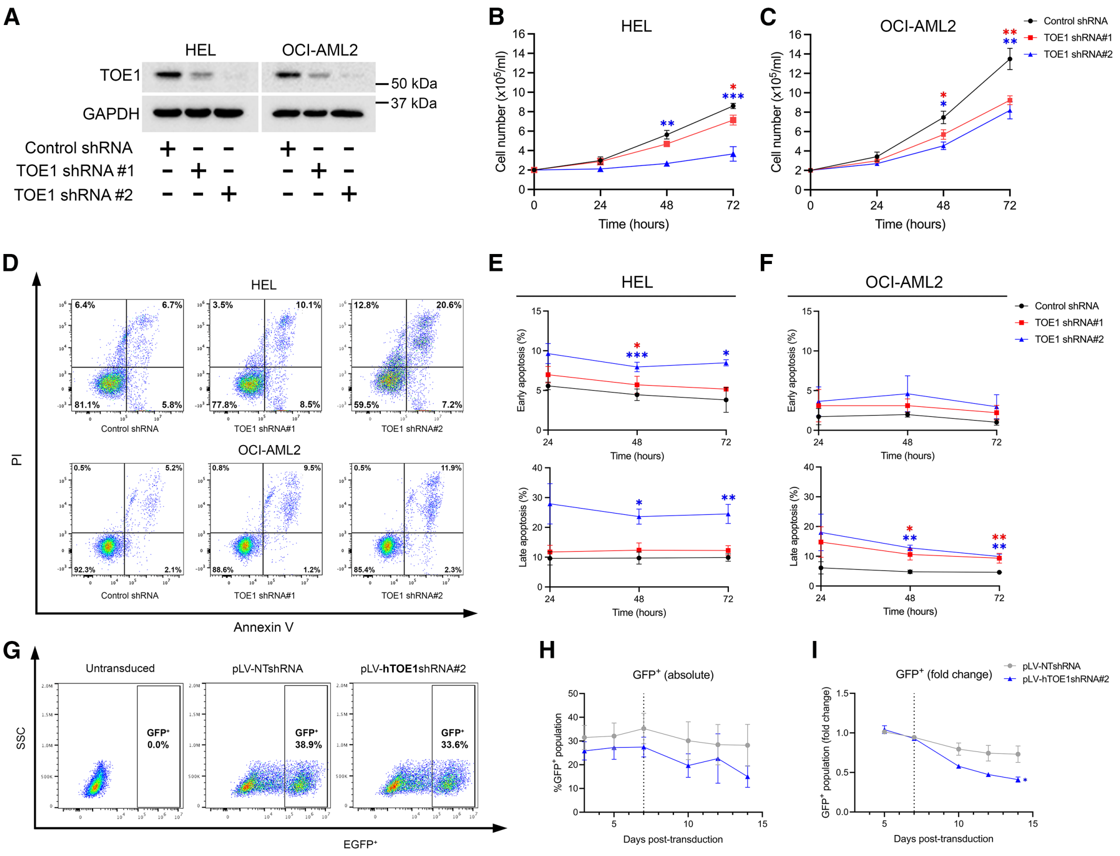

Figure 5. TOE1 impacts the proliferation and survival of AML cell lines and human CD34$^+$ HSPCs

(A) Immunoblot demonstrates the levels of TOE1 expression in HEL and OCI-AML2 cells ± TOE1 shRNA. GAPDH was utilized as the loading control. Line graphs demonstrate the impact of TOE1 depletion on growth of (B) HEL and (C) OCI-AML2 cells over 72 h of *in vitro* culture.
(D) Representative flow cytometric plots show cell survival assessed by Annexin V/PI staining in HEL and OCI-AML2 cells ± TOE1 shRNA after 48 h of *in vitro* culture. The summary data are presented as line graphs over 72 h for (E) HEL and (F) OCI-AML2 cells.
(G) Representative flow cytometric plots demonstrate the lentiviral efficiency in primary CD34$^+$ HSPCs 4 days post-transduction. The frequency of GFP$^+$ events of human cord blood-derived CD34$^+$ HSPCs transduced with non-targeting shRNA (pLV-NTshRNA) or TOE1 shRNA (pLV-hTOE1shRNA#2) 1 day following isolation, compared to matched untransduced cells. Line graphs show the (H) absolute- and (I) fold-change in the proportion of GFP$^+$ HSPCs during 14 days of *in vitro* culture post-lentiviral transduction with pLV-NTshRNA or pLV-hTOE1shRNA#2. Vertical line at day 7 post-transduction indicates switch to steady state differentiation media. Across all data error bars indicate mean ± 1SD with statistical significance denoted as *$p < 0.05$, **$p < 0.01$, and ***$p < 0.001$, $n = 3$.

initial lentiviral transduction rates of 60–98% GFP$^+$, all double-transduced cells were enriched to over 98% GFP$^+$ via FACS (Figure S8). Following selection, we next immunoblotted double-transduced cell lines to examine protein expression and confirmed sufficient TOE1 depletion alongside control or ectopic PAK2 levels, while also reaffirming PAK2 reduction upon TOE1 depletion (Figures 7D and 7E). The assessment of proliferation rates showed that single TOE1 shRNA#2 cells exhibited significantly abrogated growth rates as previously observed, which were restored to similar rates as control NT shRNA cells in the presence of ectopic PAK2 (Figure 7F). HEL/OCI-AML2 cells harboring PAK2 overexpression alone did exhibit a small but non-significant increase in proliferation rates. To see if this TOE1:PAK2 regulatory axis could extend to govern the proliferation of healthy cells, we repeated the experiment in normal human CB-derived HSPCs. We first observed that TOE1 depletion in puromycin-selected primary human HSPC cultures also resulted in PAK2 reduction (Figure 7G), indicating this regulatory axis exists in healthy HSPC. Finally, as observed in Figure 7H, the presence of ectopic PAK2 was able to overcome the previously observed growth-inhibitory impact of TOE1 depletion in human HSPC over 14 days of *in vitro* culture, and significantly enhanced proliferative expansion. Taken together, these data indicate that TOE1 regulates the proliferation of

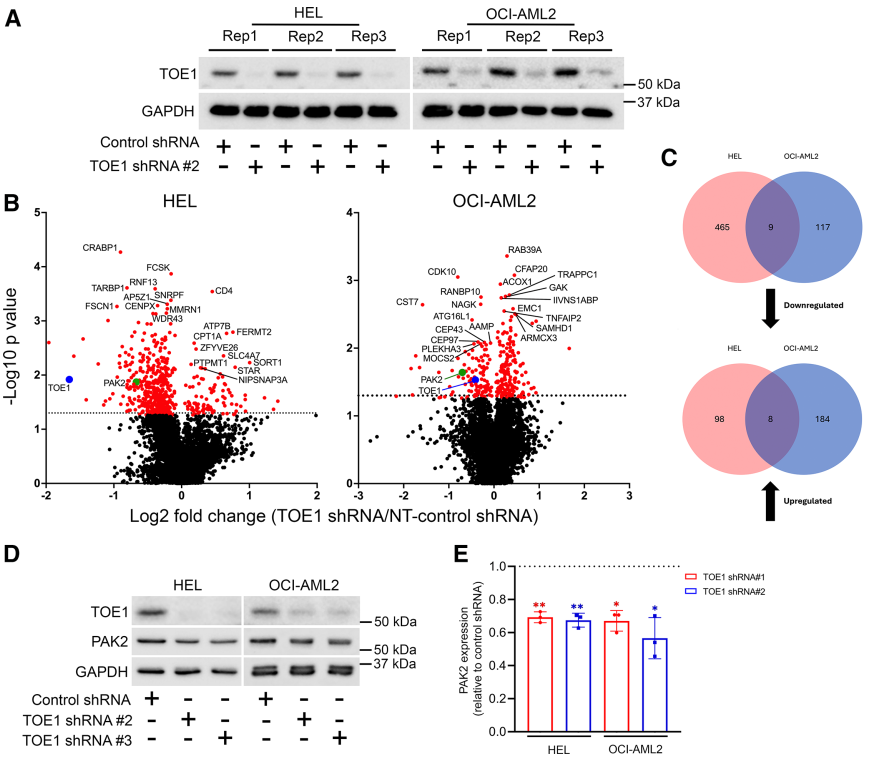

Figure 6. Proteomics analysis of TOE1-depleted AML cell lines identified PAK2 as a commonly regulated protein target

(A) Immunoblot demonstrates the levels of TOE1 expression in HEL and OCI-AML2 experimental replicates ± TOE1 shRNA prior to mass spectrometry analysis.

(B) Volcano plots show differentially expressed proteins in HEL and OCI-AML2 cells with TOE1 shRNA relative to NT-shRNA. Horizonal dashed line indicates the threshold for statistical significance at p=0.05 on $\log_{10}$ scale (=1.3). Highlighted red dots represent significantly altered peptides (p < 0.05). TOE1 is represented as a blue dot while PAK2 is highlighted by a green dot. The ten most significantly upregulated and downregulated peptides from three experimental replicates are annotated for each cell line.

(C) Venn diagrams demonstrate the total number of peptides identified to be significantly down- or upregulated in response to TOE1 knockdown, with the common peptides highlighted.

(D) Representative immunoblot shows the protein levels of PAK2 in HEL and OCI-AML2 cells ± TOE1 shRNA. GAPDH was utilized as the loading control.

(E) Fold change in PAK2 protein expression level in HEL and OCI-AML2 cells with TOE1 shRNA relative to NT-shRNA as quantified through densitometric quantification (normalized to GAPDH within each respective sample in arbitrary units). Error bars indicate mean ± 1SD, and statistical significance is denoted as *p < 0.05 and **p < 0.01, n = 3.

both normal HSPCs and leukemia cells, partly through PAK2 modulation.

DISCUSSION

This study has uncovered TOE1 as a critical regulator of human HSPC and leukemia cell growth/survival for the first time. We found protein levels to be dysregulated across primary AML samples, and higher *TOE1* expression was associated with inferior patient survival and poor-risk disease. To date, TOE1 has been associated with a highly diverse range of cellular functions, including telomere maintenance (Deng et al., 2019), p53 regulation (Sperandio et al., 2009), and viral infection (Sperandio et al., 2015), and our latest data support an emerging role in human cancer. A recent study in HCC implicated TOE1 in a drug resistance mechanism where it stabilized EGFR expression through an MYC-STAMBPL1 axis (Zhang et al., 2024). However, its action could also be context-dependent given its original characterization as a growth suppressor (De Belle et al., 2003), and more recent association as a tumor suppressor in gastric tumors through positive regulation p53/p21 expression and control of cell cycle progression (Sun et al., 2025). This is also the first report of TOE1 regulation of a human stem cell system. This could partly be through its regulation of Wnt/β-catenin signaling (LEF-1), which we also demonstrate in this paper, given that canonical Wnt signaling is known to regulate multiple mammalian stem cell systems (Nusse and Clevers, 2017). In mice, TOE1 has been shown to regulate the proliferation and differentiation of neural progenitors. Through TOE1 depletion and transcriptome analysis, the authors demonstrated differential expression of several important cell signaling components, including those from Notch, TNF and Wnt signaling (Deng et al., 2024). The importance of TOE1 to normal healthy HSPCs, and any wider stem systems beyond, could preclude its use as a therapeutically actionable target given the anticipated cytotoxicity. Regardless, there are no clinically approved direct TOE1 inhibitors even though new targeting strategies are emerging. The Cravatt group recently adopted a chemical base-editing strategy to identify essential targetable cysteines for cancer-dependent proteins (Li et al., 2023). Of the >1750 proteins identified from the DepMap Portal, 270 were defined as "strongly selective" indicating restricted dependency

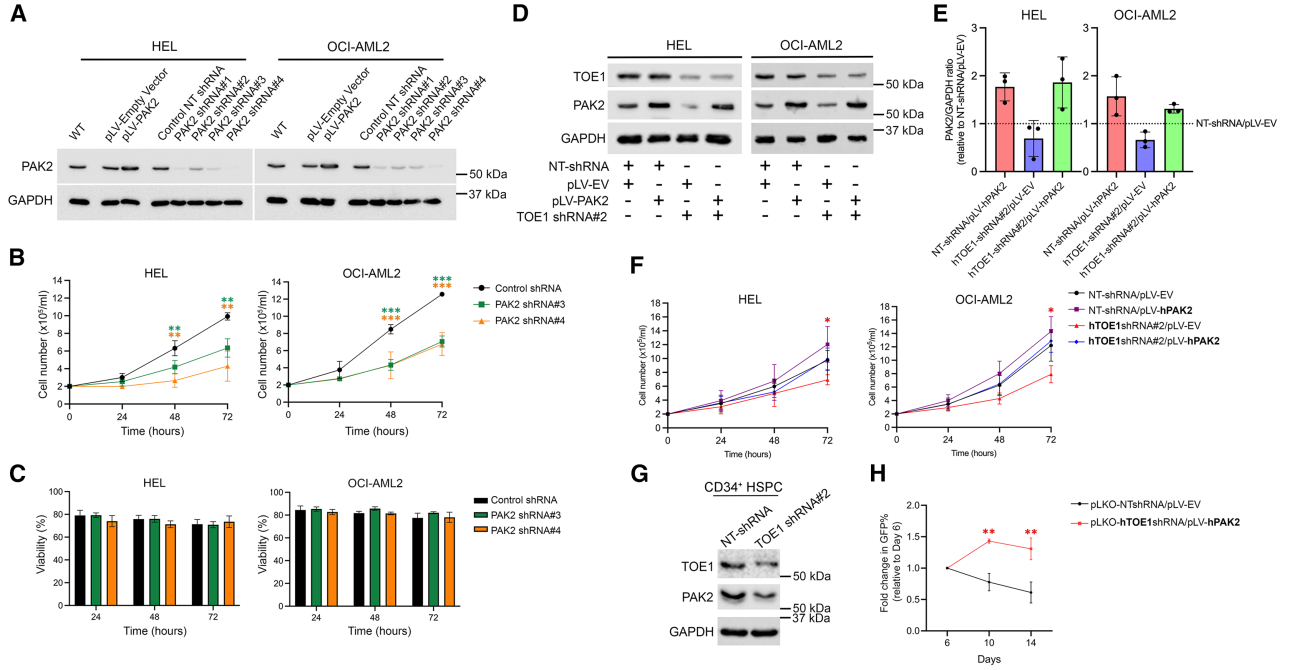

Figure 7. TOE1's growth-promoting influence in leukemic and normal hematopoietic cells is partly mediated through PAK2

(A) Immunoblot shows the levels of PAK2 in myeloid leukemia cells ± PAK2 shRNA and ± pLV-PAK2. GAPDH was utilized as the loading control.

(B and C) Graphs demonstrate the (B) growth and (C) viability (calculated via Annexin V/PI staining) of HEL and OCI-AML2 cells ± PAK2 shRNA over 72 h of *in vitro* culture.

(D) Representative immunoblot demonstrates the protein levels of TOE1 and PAK2 following double lentiviral transduction with control shRNAs (NT-shRNA or pLV-EV) alongside TOE1 shRNA#2 or pLV-PAK2 and post-FACS purification of GFP$^+$ cells.

(E) Fold-change summary of PAK2 protein expression level in HEL and OCI-AML2 cells double transduced with NT-shRNA/pLV-PAK2, TOE1 shRNA#2/pLV-EV or TOE1 shRNA#2/pLV-PAK2 relative to double control NT-shRNA/pLV-EV, as quantified through densitometric quantification (normalized to GAPDH within each respective sample in arbitrary units).

(F) Summary graphs demonstrate the growth of double transduced HEL and OCI-AML2 over 72 h of *in vitro* culture.

(G) Representative immunoblot demonstrates the expression of TOE1 and PAK2 in CD34$^+$ HSPC ± TOE1 shRNA.

(H) Line graph shows the fold change in percentage GFP$^+$ cells in CB-derived CD34$^+$ HSPC double lentiviral transduced with either NTshRNA/pLV-EV control plasmids or hTOE1shRNA#2/pLV-hPAK2 plasmids and cultured *in vitro* for 14 days. Error bars indicate mean ± 1SD, with statistical significance denoted as $^*p < 0.05$, $^{**}p < 0.01$, and $^{***}p < 0.001$, $n = 3$.

in specific cancer cell lines. Of a 12-cell line cohort, the growth of MCC142 (Merkel cell carcinoma) and PANC1005 (pancreatic adenocarcinoma) cells was found to be highly TOE1-dependent, extending the number/type of cancers where TOE1 may serve oncogenic function. From here, the authors developed two molecules (WX-02–33 or WX-02–13) targeting Cys80 of TOE1, of which WX-02–33 was found to allosterically inhibit the nuclease activity of TOE1 (while promoting TOE1 binding to spliceosome complexes) and perturb the growth of MCC142 cells in competition assays. Using WX-02–33 as a reference point, a more recent study by Kehinde et al., explored TOE1 Cys80 targeted molecules further with the development of compound 0462, which exhibited more favorable interaction profiles, covalent binding dynamics, free binding energetics, and per-residue energy contributions (Kehinde et al., 2024). These studies suggest direct TOE1 targeting could be pharmacologically viable should a suitable and safe therapeutic window be identified.

Our original motivation for investigating TOE1's role in a hematopoietic setting came from its significant enrichment in the β-catenin interactome of multiple myeloid cell lines, which was subsequently validated through reciprocal TOE1 Co-IPs in cell lines and a primary AML patient sample in this study. Despite this robust RNA-independent interaction, we were unable to demonstrate any substantial impact of TOE1 modulation on the level or localization of β-catenin itself. We intended to assess the impact of β-catenin on TOE1's RNA editing/interaction capacity; however, we were unable to isolate any detectable RNA through TOE1 RIP/CLIP assessment, which was surprising given TOE1's well-established role as an

exonuclease/deadenylase for short nuclear non-coding RNAs (Deng et al., 2019; Fong et al., 2013; Lardelli and Lykke-Andersen, 2020; Lardelli et al., 2017; Ma et al., 2024; Son et al., 2018; Wagner et al., 2007). It is likely these TOE1:snRNAs interactions are too transient to be reliably detected through traditional RIP/CLIP approaches, requiring a more sophisticated approach such as HyperTRIBE (Nguyen et al., 2020), or a catalytically dead TOE1 variant, to identify TOE1 RNA targets in β-catenin modulated cells. Instead, we opted to focus on the one consistent area of crosstalk we were able to identify between TOE1 and Wnt activity, which was through LEF-1 regulation. We observed no alteration to the overall *LEF1* transcript level and instead found TOE1 altered the stability of LEF-1 protein as assessed by a CHX chase. Given TOE1's role in regulating snRNA maturation (Huynh and Parker, 2023), we cannot eliminate the possibility that the reduced LEF-1 protein stability upon TOE1 loss is not also due to unstable *LEF1* mRNA. Indeed, the MYC-STAMBPL1-TOE1 axis which was reported to regulate EGFR expression in HCC, appeared to impact both transcript and protein levels of EGFR, which contributed to Lenvatinib resistance (Zhang et al., 2024). Part of this mechanism was proposed to be through the STAMBPL1's deubiquitination of TOE1, preventing its lysosomal degradation. However, our experiments in leukemia cells were unable to demonstrate any strong regulation of TOE1 protein in response to autolysosomal/proteasomal inhibition, where it remained remarkably stable. Therefore, the mechanism through which TOE1 regulates the LEF-1 stability remains elusive.

Regardless, LEF-1 alone could not universally explain the reduced growth/survival observed upon TOE1 depletion in AML cell lines/HSPC, since OCI-AML2 does not express LEF-1. To address this, we assessed global protein abundance in TOE1-depleted leukemia cells for the first time using quantitative MS. From this analysis, we observed only moderate alterations to global peptide abundance in response to TOE1 depletion. This could possibly be due to incomplete TOE1 ablation, and/or compensation by the functional homolog PARN, which we also found to be abundant in myeloid cells (Huynh and Parker, 2023; Son et al., 2018). Throughout our study, we found the degree of TOE1 depletion correlated with resulting phenotypic or genotypic regulation. This is mirrored with DepMap Portal data, where CRISPR/Cas9-mediated TOE1 depletion is more detrimental to cell growth/survival than RNAi, presumably due to superior TOE1 ablation. Nevertheless, using shRNA, we were able to identify significant and consistent alterations to several proteins across both TOE1-depleted AML cell lines, including PAK2. PAK2 was reduced by TOE1 depletion in multiple AML cell lines and primary *in vitro* cultured HSPCs. PAKs are a family of important evolutionarily conserved serine/threonine kinases implicated in numerous critical cellular processes, including cytoskeletal arrangement, motility, apoptosis, proliferation, and cell division (Kumar et al., 2006; Rane and Minden, 2014; Zhao and Manser, 2012). PAK2 was prioritized for further study given its previous association with several processes important to murine HSPC biology, including homing/migration (Reddy et al., 2016), and more importantly for this study, proliferation. HSPCs from conditional PAK2 knockout mice exhibited growth and survival defects in multi-cytokine-supplemented *in vitro* culture, albeit with no difference in self-renewal capacity (Zeng et al., 2015), a similar phenotype we observed with TOE1 shRNA in human HSPCs. Elsewhere, PAK2 has been implicated in hematological malignancies (Wu and Jiang, 2022), and further human cancers beyond (Chen et al., 2025). Whereas PAK1 and 4 are the predominant isoforms deregulated in solid tumors (Kumar et al., 2006), PAK1 and 2 are key drivers of BCR-ABL^{+} chronic myeloid leukemia cells. PAK2, rather than PAK1, was a key regulator of CML cell growth, but only a limited impact on survival was observed until PAK1 and 2 were ablated in combination (Edlinger et al., 2017). No PAK2-specific inhibitors have been developed yet; however, pan-PAK inhibitors such as PF-3758309, FRAX-486, and IPA-3 are available and have shown promise in lymphoid malignancies (Chung et al., 2019; Siekmann et al., 2018; Su et al., 2023) and AML (Casado et al., 2025). However, pre-existing reports alongside our latest study suggest a very carefully defined therapeutic window is necessary, given the likely HSPC toxicity.

Although PAK2 was shown to contribute to human HSPC proliferation and indeed AML cell growth more generally, it could only partially recover the proliferative defect induced upon TOE1 loss, and ectopic PAK2 expression did not significantly impact cell survival. This implies that TOE1 may influence cell proliferation/viability through other mechanisms not explored in this study. TOE1 was originally discovered through its ability to modulate EGR1-mediated growth (De Belle et al., 2003), and has since been shown to interact with p53 and influence cell growth by modulating its transcriptional activity through targets such as p21 (Sperandio et al., 2009). Interestingly, we found *TP53* mutations were the most significantly enriched gene mutation in patients with TOE1^{high} AML, potentially explaining the poor risk/reduced survival linked with TOE1 and indicating a wider regulatory axis worthy of further exploration in AML. Elsewhere, TOE1 influenced NOTCH and TNF signaling in neural progenitors (Deng et al., 2024), and more recently was shown to promote HCC proliferation and metastasis through Hippo signaling (Ao et al., 2025).

In summary, this study has shown for the first time that the β-catenin-interacting protein TOE1 impacts Wnt signaling in leukemia cells through LEF-1 modulation, and its levels are dysregulated in leukemia, where it regulates the proliferation of human AML cells and HSPC through PAK2.

METHODS

Primary samples

Bone marrow, peripheral blood, or leukapheresis samples from patients diagnosed with AML (clinical information provided in Table S1) were collected in accordance with the Declaration of Helsinki and with approvals of University Hospitals Bristol and Weston NHS Foundation Trust and the London Brent Research Ethics Committee (12/LO/1193). Human CB was obtained following informed consent from healthy mothers at full-term undergoing elective cesarean sections at Royal Sussex County Hospital and Princess Royal Hospital, with approval from University Hospitals Sussex NHS Foundation Trust, the East of England-Essex Research Ethics Committee, HRA, and Health and Care Research Wales (18/EE/0403). Extraction and purification of mononuclear cells (MNCs) and/or $CD34^+$ HSPC is outlined in the supplemental information.

Cell culture and drug treatments

Primary and immortalized cells were maintained as outlined in supplemental information. Additionally, following 7 days of liquid culture and post-lentiviral transduction, primary HSPC cultures were switched from expansion media into steady-state differentiation media containing 20ng/mL of SCF, 5ng/mL of IL3, and G-CSF (Proteintech). β-Catenin was stabilized using 5 μM of the GSK3β inhibitor CHIR99021 (Merck-Millipore) overnight. Proteasomal turnover and autophagy were inhibited through 1 μM MG132 (Merck-Millipore) and 100nM Bafilomycin A1 (Merck-Millipore), respectively, while translation was inhibited with 100nM CHX (Merck-Millipore).

Whole-cell lysis

Cells ($2–5\times10^6$) were washed in ice-cold PBS and resuspended in 100μL 1× lysis buffer (Cell Signaling Technology, Leiden, Netherlands) containing Complete Mini Protease Inhibitor cocktail (Roche, Welwyn Garden City, Hertfordshire) and incubated for 30min with occasional vortexing (Merck-Millipore) to maximize lysis. Insoluble material was removed by centrifugation at 21,000x*g* for 10 min, and the resulting homogenate was stored at −80°C until further use.

RT-qPCR

RNA was extracted with the Zymo RNA Miniprep kit (Cambridge Bioscience, Cambridge, Cambridgeshire), subjected to DNase treatment with TURBO DNase (Thermo Fisher Scientific, Altrincham, Cheshire), and cleaned with the Monarch RNA Cleanup kit (New England BioLabs, Hitchin, Hertfordshire) according to the manufacturer's instructions. All primers used in the study are listed in Table S2 and were optimized to show efficiency between 90 and 110% using an SYBR-green kit (Apto-Gen, London, UK). RT-qPCR programs used as per manufacturer's instructions (Merck-Millipore). RT-qPCR analysis was performed on QuantStudio 3 Real-Time PCR System (Thermo Fisher Scientific) and analyzed on Design & Analysis 2 (Thermo Fisher Scientific). Relative gene expression was calculated with the ΔΔCT method with *GAPDH* as the reference gene.

Proteomics

For TMT labeling, chromatography, MS, and data analysis, consult the supplemental information.

Immunoblotting

Immunoblotting was performed as described previously (Morgan et al., 2019), with antibodies to β-catenin, TOE1, PARN, LEF-1, TCF-4, Lamin A/C, α-tubulin, β-actin, and GAPDH, details listed in Table S3). Densitometric analysis of LEF-1 expression was performed utilizing ImageJ software version 1.54 (National Institute of Health, Bethesda, Maryland, USA) normalizing to the GAPDH density present within each sample.

Lentivirus generation and transduction

Replication-deficient lentiviral particles were generated as outlined in the supplemental methods using the expression plasmids outlined in Table S4.

Flow cytometry

For Wnt reporter assessment, 2×10^5 BAR/fuBAR containing cells were treated overnight with either DMSO or CHIR99021. TCF/LEF reporter activity (Venus Yellow Fluorescent intensity) was assessed through CytoFLEX Flow cytometer (Beckman Coulter, Amersham, Buckinghamshire) and analyzed with FlowJo software version 10.8.2 (Tree Star Inc., Ashland, OR). For immunophenotyping, up to 5×10^4 CB-derived $CD34^+$ HSPCs were resuspended in 100μL of staining buffer (1× OBS, 0.5% BSA) containing 10μg/mL CD34-PE, CD45-PerCPCy5.5, CD36-PE, and CD13-PerCPCy5.5 (all Biolegend, London, UK) or the equivalent concentration-, manufacturer-, and isotype-matched control antibodies. 7-AAD (Thermo Fisher Scientific) was utilized to exclude non-viable cells according to the manufacturer's instructions.

Annexin V/PI assay

The Annexin V Apoptosis Detection Kit (BD Pharmingen) was utilized to quantify the following sub-populations: viable/live (Annexin V^{neg}, PI^{neg}), early apoptotic (Annexin V^{pos}, PI^{neg}), late apoptotic ($Annexin^{pos}$, PI^{pos}), and necrotic/dead cells (Annexin V^{neg}, PI^{pos}).

Immunofluorescence (IF)

2×10^6 cells of interest were washed with PBS and resuspended in 1mL of 2% paraformaldehyde and incubated for 20 min at room temperature with agitation. The cells were subsequently resuspended in 1mL quenching buffer (100mM glycine in PBS) and 1mL of permeabilization buffer (0.1% Triton TX100 in PBS) with further washes with staining buffer (1× OBS, 0.5% BSA). The primary antibodies utilized for immunofluorescence (IF) were: anti-TOE1 (Proteintech; Bethyl Laboratories, Montgomery, TX), anti-β-catenin (BD Biosciences, Wokingham, Berkshire). The secondary antibodies utilized were: goat anti-mouse Alexa Fluor 488 and goat anti-rabbit Alexa Fluor 647 (Invitrogen, Paisley, Renfrewshire). DAPI was utilized as the nuclear counterstain. Images were captured on the Zeiss LSM 880 confocal microscope utilizing a 63× oil immersion objective with the Zen (Black Edition) software (version 2.3).

RIP/CLIP

RIP/CLIP assays were performed as previously (Wagstaff et al., 2025).

Nuclear/cytoplasmic fractionation

Nuclear/cytoplasmic fractionation was performed as previously (Morgan et al., 2019).

Statistics

A one-sample or Student's *t* test was utilized to analyze differences between control and modulated gene expression conditions, utilizing three technical replicates within three experimental replicate samples, unless otherwise stated. Immunoblot/gel images presented are representative of three experimental replicates unless otherwise stated. All graphs were prepared utilizing GraphPad Prism (software version 8.0.1; GraphPad, Boston, MA). All data are expressed as the mean ±1 standard deviation (SD) unless otherwise stated.

RESOURCE AVAILABILITY

Lead contact

Requests for further information and resources should be directed to and will be fulfilled by the lead contact, Rhys Morgan (rhys.morgan@sussex.ac.uk).

Materials availability

All reagents generated in this study are available from the lead contact without restriction.

Data and code availability

All data reported in this paper will be shared by the lead contact upon request. This study does not report original code. Raw MS data from TOE1-depleted AML cells are available via ProteomeXchange with identifier PXD070891. Any additional information required to reanalyze the data reported in this paper is available from the lead contact upon request.

ACKNOWLEDGMENTS

This work was funded by the Kay Kendall Leukaemia Fund (RM: KKL1051/KKL1446), Leukaemia & Myeloma Research UK (RM: 4–5/06.21R), Children's Cancer and Leukaemia Group (CCLGA 2023 16 Morgan), British Society for Haematology (HP: 44299), the Sussex Cancer Fund (HP) and the Republic of Türkiye Ministry of National Education (OS). This work was also funded by a Biotechnology and Biological Sciences Research Council grant to SFN and BPT (BB/V001701/1). Thanks to Dr. Paraskevi Diamanti (University of Bristol) for AML patient sample collection and Lizzy Hoole (Institute for Child Life & Health, University Hospitals Bristol & Weston NHS Foundation Trust) for supplying clinical data. Thanks to the midwives and clinical research nurses at University Hospitals Sussex (UHS) NHS trust, including Raquel Akieme, Valentina Toska, Lorraine Shah-Goodwin, Denise Skinner, Carla Clegg, Edina Lalu, and Elohor Uwadiogbu, for the collection of human umbilical cord blood. We thank all the technicians in the School of Life Sciences who maintain our laboratories and facilities. We are indebted to the patients and their families who gave consent for their samples to be used for our research.

AUTHOR CONTRIBUTIONS

HP and OS performed experiments, analyzed data, and co-wrote the manuscript. MW executed experiments and provided laboratory support, while AG and DP optimized CLIP assays. BK assisted with cord blood collection/processing, KH performed TMT-LC/MS analysis, and AB provided primary AML samples. SN, ELM, and BT provided experimental guidance, equipment, and reagents. TJC and RGM performed experiments, analyzed data, co-wrote the manuscript, secured funding, and directed the study.

DECLARATION OF INTERESTS

All the authors have no competing interests.

SUPPLEMENTAL INFORMATION

Supplemental information can be found online at https://doi.org/10.1016/j.stemcr.2026.102894.

Received: December 23, 2025
Revised: March 24, 2026
Accepted: March 24, 2026
Published: April 23, 2026

REFERENCES

Ao, J.Y., Cai, Y., Xu, S.T., Yang, B.Y., Sun, X., and Shi, Y. (2025). Transcriptomic analysis reveals the potential role of TOE1 in hepatocellular carcinoma. Sci. Rep. *16*, 69. https://doi.org/10.1038/s41598-025-29408-1.

Casado, P., Marfa, S., Hadi, M.M., Gerdes, H., Martin-Guerrero, S.M., Miraki-Moud, F., Rajeeve, V., and Cutillas, P.R. (2025). Phosphoproteomics identifies determinants of PAK inhibitor sensitivity in leukaemia cells. Cell Commun. Signal. *23*, 135. https://doi.org/10.1186/s12964-025-02107-0.

Cerami, E., Gao, J., Dogrusoz, U., Gross, B.E., Sumer, S.O., Aksoy, B.A., Jacobsen, A., Byrne, C.J., Heuer, M.L., Larsson, E., et al. (2012). The cBio cancer genomics portal: an open platform for exploring multidimensional cancer genomics data. Cancer Discov. *2*, 401–404. https://doi.org/10.1158/2159-8290.CD-12-0095.

Chen, X.P., Yang, Z.T., Yang, S.X., Li, E.M., and Xie, L. (2025). PAK2 as a therapeutic target in cancer: Mechanisms, challenges, and future perspectives. Biochim. Biophys. Acta. Rev. Cancer *1880*, 189246. https://doi.org/10.1016/j.bbcan.2024.189246.

Chung, E.Y., Mai, Y., Shah, U.A., Wei, Y., Ishida, E., Kataoka, K., Ren, X., Pradhan, K., Bartholdy, B., Wei, X., et al. (2019). PAK Kinase Inhibition Has Therapeutic Activity in Novel Preclinical Models of Adult T-Cell Leukemia/Lymphoma. Clin. Cancer Res. *25*, 3589–3601. https://doi.org/10.1158/1078-0432.CCR-18-3033.

De Belle, I., Wu, J.X., Sperandio, S., Mercola, D., and Adamson, E.D. (2003). In vivo cloning and characterization of a new growth suppressor protein TOE1 as a direct target gene of Egr1. J. Biol. Chem. *278*, 14306–14312. https://doi.org/10.1074/jbc.M210502200.

de Bruijn, I., Kundra, R., Mastrogiacomo, B., Tran, T.N., Sikina, L., Mazor, T., Li, X., Ochoa, A., Zhao, G., Lai, B., et al. (2023). Analysis and Visualization of Longitudinal Genomic and Clinical Data from the AACR Project GENIE Biopharma Collaborative in cBioPortal. Cancer Res. *83*, 3861–3867. https://doi.org/10.1158/0008-5472.Can-23-0816.

Deng, T., Huang, Y., Weng, K., Lin, S., Li, Y., Shi, G., Chen, Y., Huang, J., Liu, D., Ma, W., and Songyang, Z. (2019). TOE1 acts as a 3′ exonuclease for telomerase RNA and regulates telomere maintenance. Nucleic Acids Res. *47*, 391–405. https://doi.org/10.1093/nar/gky1019.

Deng, T., Wu, X., Wang, Y., Fan, X., and Hu, B. (2024). Toe1 promotes proliferation and differentiation of neural progenitor cells. Heliyon *10*, e39535. https://doi.org/10.1016/j.heliyon.2024.e39535.

Dietrich, P.A., Yang, C., Leung, H.H.L., Lynch, J.R., Gonzales, E., Liu, B., Haber, M., Norris, M.D., Wang, J., and Wang, J.Y. (2014). GPR84 sustains aberrant beta-catenin signaling in leukemic stem cells for maintenance of MLL leukemogenesis. Blood *124*, 3284–3294. https://doi.org/10.1182/blood-2013-10-532523.

Döhner, H., Wei, A.H., and Löwenberg, B. (2021). Towards precision medicine for AML. Nat. Rev. Clin. Oncol. *18*, 577–590. https://doi.org/10.1038/s41571-021-00509-w.

Edlinger, L., Berger-Becvar, A., Menzl, I., Hoermann, G., Greiner, G., Grundschober, E., Bago-Horvath, Z., Al-Zoughbi, W., Hoefler, G., Brostjan, C., et al. (2017). Expansion of BCR/ABL1(+) cells requires PAK2 but not PAK1. Br. J. Haematol. *179*, 229–241. https://doi.org/10.1111/bjh.14833.

Fong, C.Y., Gilan, O., Lam, E.Y.N., Rubin, A.F., Ftouni, S., Tyler, D., Stanley, K., Sinha, D., Yeh, P., Morison, J., et al. (2015). BET inhibitor resistance emerges from leukaemia stem cells. Nature *525*, 538–542. https://doi.org/10.1038/nature14888.

Fong, K.W., Li, Y., Wang, W., Ma, W., Li, K., Qi, R.Z., Liu, D., Songyang, Z., and Chen, J. (2013). Whole-genome screening identifies proteins localized to distinct nuclear bodies. J. Cell Biol. *203*, 149–164. https://doi.org/10.1083/jcb.201303145.

Gao, J., Aksoy, B.A., Dogrusoz, U., Dresdner, G., Gross, B., Sumer, S.O., Sun, Y., Jacobsen, A., Sinha, R., Larsson, E., et al. (2013). Integrative analysis of complex cancer genomics and clinical profiles using the cBioPortal. Sci. Signal. *6*, pl1. https://doi.org/10.1126/scisignal.2004088.

Halik, A., Arends, C.M., Bullinger, L., Damm, F., and Frick, M. (2022). Refining AML Treatment: The Role of Genetics in Response and Resistance Evaluation to New Agents. Cancers (Basel) *14*, 1689. https://doi.org/10.3390/cancers14071689.

Huynh, T.N., and Parker, R. (2023). The PARN, TOE1, and USB1 RNA deadenylases and their roles in non-coding RNA regulation. J. Biol. Chem. *299*, 105139. https://doi.org/10.1016/j.jbc.2023.105139.

Jakobi, R., McCarthy, C.C., Koeppel, M.A., and Stringer, D.K. (2003). Caspase-activated PAK-2 is regulated by subcellular targeting and proteasomal degradation. J. Biol. Chem. *278*, 38675–38685. https://doi.org/10.1074/jbc.M306494200.

Kehinde, I.O., Oduro-Kwateng, E., and Soliman, M.E.S. (2024). Allosteric covalent inhibition of TOE1 as potential unexplored anti-cancer target: structure-based virtual screening and covalent molecular dynamics analysis. J. Recept. Signal Transduct. Res. *44*, 97–106. https://doi.org/10.1080/10799893.2024.2411690.

Kim, I., Kwak, H., Lee, H.K., Hyun, S., and Jeong, S. (2012). beta-Catenin recognizes a specific RNA motif in the cyclooxygenase-2 mRNA 3′-UTR and interacts with HuR in colon cancer cells. Nucleic Acids Res. *40*, 6863–6872. https://doi.org/10.1093/nar/gks331.

Kumar, R., Gururaj, A.E., and Barnes, C.J. (2006). p21-activated kinases in cancer. Nat. Rev. Cancer *6*, 459–471. https://doi.org/10.1038/nrc1892.

Lardelli, R.M., and Lykke-Andersen, J. (2020). Competition between maturation and degradation drives human snRNA 3′ end quality control. Genes Dev. *34*, 989–1001. https://doi.org/10.1101/gad.336891.120.

Lardelli, R.M., Schaffer, A.E., Eggens, V.R.C., Zaki, M.S., Grainger, S., Sathe, S., Van Nostrand, E.L., Schlachetzki, Z., Rosti, B., Akizu, N., et al. (2017). Biallelic mutations in the 3′ exonuclease TOE1 cause pontocerebellar hypoplasia and uncover a role in snRNA processing. Nat. Genet. *49*, 457–464. https://doi.org/10.1038/ng.3762.

Lee, H.K., and Jeong, S. (2006). Beta-Catenin stabilizes cyclooxygenase-2 mRNA by interacting with AU-rich elements of 3′-UTR. Nucleic Acids Res. *34*, 5705–5714. https://doi.org/10.1093/nar/gkl698.

Li, H., Ma, T., Remsberg, J.R., Won, S.J., DeMeester, K.E., Njomen, E., Ogasawara, D., Zhao, K.T., Huang, T.P., Lu, B., et al. (2023). Assigning functionality to cysteines by base editing of cancer dependency genes. Nat. Chem. Biol. *19*, 1320–1330. https://doi.org/10.1038/s41589-023-01428-w.

Luis, T.C., Ichii, M., Brugman, M.H., Kincade, P., and Staal, F.J.T. (2012a). Wnt signaling strength regulates normal hematopoiesis and its deregulation is involved in leukemia development. Leukemia *26*, 414–421. https://doi.org/10.1038/leu.2011.387.

Luis, T.C., Killmann, N.M.B., and Staal, F.J.T. (2012b). Signal transduction pathways regulating hematopoietic stem cell biology: Introduction to a series of Spotlight Reviews. Leukemia *26*, 86–90.

Ma, T., Xiong, E.S., Lardelli, R.M., and Lykke-Andersen, J. (2024). Sm complex assembly and 5′ cap trimethylation promote selective processing of snRNAs by the 3′ exonuclease TOE1. Proc. Natl. Acad. Sci. USA *121*, e2315259121. https://doi.org/10.1073/pnas.2315259121.

Mellacheruvu, D., Wright, Z., Couzens, A.L., Lambert, J.P., St-Denis, N.A., Li, T., Miteva, Y.V., Hauri, S., Sardiu, M.E., Low, T.Y., et al. (2013). The CRAPome: a contaminant repository for affinity purification-mass spectrometry data. Nat. Methods *10*, 730–736. https://doi.org/10.1038/nmeth.2557.

Morgan, R.G., Pearn, L., Liddiard, K., Pumford, S.L., Burnett, A.K., Tonks, A., and Darley, R.L. (2013). γ-Catenin is overexpressed in acute myeloid leukemia and promotes the stabilization and nuclear localization of β-catenin. Leukemia *27*, 336–343. https://doi.org/10.1038/leu.2012.221.

Morgan, R.G., Ridsdale, J., Payne, M., Heesom, K.J., Wilson, M.C., Davidson, A., Greenhough, A., Davies, S., Williams, A.C., Blair, A., et al. (2019). LEF-1 drives aberrant beta-catenin nuclear localization in myeloid leukemia cells. Haematologica *104*, 1365–1377. https://doi.org/10.3324/haematol.2018.202846.

Nguyen, D.T.T., Lu, Y., Chu, E.L., Yang, X., Park, S.M., Choo, Z.N., Chin, C.R., Prieto, C., Schurer, A., Barin, E., et al. (2020). HyperTRIBE uncovers increased MUSASHI-2 RNA binding activity and differential regulation in leukemic stem cells. Nat. Commun. *11*, 2026. https://doi.org/10.1038/s41467-020-15814-8.

Nusse, R., and Clevers, H. (2017). Wnt/beta-Catenin Signaling, Disease, and Emerging Therapeutic Modalities. Cell *169*, 985–999. https://doi.org/10.1016/j.cell.2017.05.016.

Perry, J.M., Tao, F., Roy, A., Lin, T., He, X.C., Chen, S., Lu, X., Nemechek, J., Ruan, L., Yu, X., et al. (2020). Overcoming Wnt-beta-catenin dependent anticancer therapy resistance in leukaemia stem cells. Nat. Cell Biol. *22*, 689–700. https://doi.org/10.1038/s41556-020-0507-y.

Rane, C.K., and Minden, A. (2014). P21 activated kinases: structure, regulation, and functions. Small GTPases *5*, e28003. https://doi.org/10.4161/sgtp.28003.

Reddy, P.N.G., Radu, M., Xu, K., Wood, J., Harris, C.E., Chernoff, J., and Williams, D.A. (2016). p21-activated kinase 2 regulates HSPC cytoskeleton, migration, and homing via CDC42 activation and interaction with beta-Pix. Blood *127*, 1967–1975. https://doi.org/10.1182/blood-2016-01-693572.

Reya, T., Duncan, A.W., Ailles, L., Domen, J., Scherer, D.C., Willert, K., Hintz, L., Nusse, R., and Weissman, I.L. (2003). A role for Wnt signalling in self-renewal of haematopoietic stem cells. Nature *423*, 409–414.

Rudel, T., and Bokoch, G.M. (1997). Membrane and morphological changes in apoptotic cells regulated by caspase-mediated activation of PAK2. Science *276*, 1571–1574. https://doi.org/10.1126/science.276.5318.1571.

Sevim, O., Park, H., and Morgan, R.G. (2025). Post-transcriptional control of gene expression by beta-catenin: expanding the non-canonical ARMoury. Oncogene *44*, 2453–2459. https://doi.org/10.1038/s41388-025-03470-5.

Shao, J., Yan, Y., Ding, D., Wang, D., He, Y., Pan, Y., Yan, W., Kharbanda, A., Li, H.Y., and Huang, H. (2021). Destruction of DNA-Binding Proteins by Programmable Oligonucleotide PROTAC (O'PROTAC): Effective Targeting of LEF1 and ERG. Adv. Sci. *8*, e2102555. https://doi.org/10.1002/advs.202102555.

Shooshtarizadeh, P., Helness, A., Vadnais, C., Brouwer, N., Beauchemin, H., Chen, R., Bagci, H., Staal, F.J.T., Coté, J.F., and Möröy, T. (2019). Gfi1b regulates the level of Wnt/beta-catenin signaling in hematopoietic stem cells and megakaryocytes. Nat. Commun. *10*, 1270. https://doi.org/10.1038/s41467-019-09273-z.

Siekmann, I.K., Dierck, K., Prall, S., Klokow, M., Strauss, J., Buhs, S., Wrzeszcz, A., Bockmayr, M., Beck, F., Trochimiuk, M., et al. (2018). Combined inhibition of receptor tyrosine and p21-activated kinases as a therapeutic strategy in childhood ALL. Blood Adv. *2*, 2554–2567. https://doi.org/10.1182/bloodadvances.2018020693.

Son, A., Park, J.E., and Kim, V.N. (2018). PARN and TOE1 Constitute a 3′ End Maturation Module for Nuclear Non-coding RNAs. Cell Rep. *23*, 888–898. https://doi.org/10.1016/j.celrep.2018.03.089.

Sperandio, S., Barat, C., Cabrita, M.A., Gargaun, A., Berezovski, M.V., Tremblay, M.J., and de Belle, I. (2015). TOE1 is an inhibitor of HIV-1 replication with cell-penetrating capability. Proc. Natl. Acad. Sci. USA *112*, E3392–E3401. https://doi.org/10.1073/pnas.1500857112.

Sperandio, S., Tardito, S., Surzycki, A., Latterich, M., and de Belle, I. (2009). TOE1 interacts with p53 to modulate its transactivation potential. FEBS Lett. *583*, 2165–2170. https://doi.org/10.1016/j.febslet.2009.06.004.

Su, N., Fang, Y., Chen, X., Chen, X., Xia, Z., Huang, H., Xia, Y., Liu, P., Tian, X., and Cai, Q. (2023). Targeting P21-activated kinase suppresses proliferation and enhances chemosensitivity in T-cell lymphoblastic lymphoma. Blood Sci. *5*, 249–257. https://doi.org/10.1097/BS9.0000000000000169.

Sun, X.L., Song, H.X., Li, J.H., Liu, Y.J., Wang, X.Y., and Zhang, L.N. (2025). TOE1 deadenylase inhibits gastric cancer cell proliferation by regulating cell cycle progression. Biochim. Biophys. Acta. Gen. Subj. *1869*, 130736. https://doi.org/10.1016/j.bbagen.2024.130736.

Vilas, G.L., Corvi, M.M., Plummer, G.J., Seime, A.M., Lambkin, G.R., and Berthiaume, L.G. (2006). Posttranslational myristoylation of caspase-activated p21-activated protein kinase 2 (PAK2) potentiates late apoptotic events. Proc. Natl. Acad. Sci. USA *103*, 6542–6547. https://doi.org/10.1073/pnas.0600824103.

Wagner, E., Clement, S.L., and Lykke-Andersen, J. (2007). An unconventional human Ccr4-Caf1 deadenylase complex in nuclear cajal bodies. Mol. Cell Biol. *27*, 1686–1695. https://doi.org/10.1128/MCB.01483-06.

Wagstaff, M., Coke, B., Hodgkiss, G.R., and Morgan, R.G. (2022). Targeting beta-catenin in acute myeloid leukaemia: past, present, and future perspectives. Biosci. Rep. *42*, BSR20211841. https://doi.org/10.1042/BSR20211841.

Wagstaff, M., Sevim, O., Goff, A., Raynor, M., Park, H., Mancini, E.J., Nguyen, D.T.T., Chevassut, T., Blair, A., Castellano, L., et al. (2025). β-Catenin interacts with canonical RBPs including MSI2 to associate with a Wnt signalling mRNA network in myeloid leukaemia cells. Oncogene *44*, 2490–2503. https://doi.org/10.1038/s41388-025-03415-y.

Wagstaff, M., Tsaponina, O., Caalim, G., Greenfield, H., Milton-Harris, L., Mancini, E.J., Blair, A., Heesom, K.J., Tonks, A., Darley, R.L., et al. (2023). Crosstalk between beta-catenin and WT1 signaling activity in acute myeloid leukemia. Haematologica *108*, 283–289. https://doi.org/10.3324/haematol.2021.280294.

Wang, C., Ge, Y., Li, R., He, G., and Lin, Y. (2023). Novel compound heterozygous missense variants in TOE1 gene associated with pontocerebellar hypoplasia type 7. Gene *862*, 147250. https://doi.org/10.1016/j.gene.2023.147250.

Wang, Y., Krivtsov, A.V., Sinha, A.U., North, T.E., Goessling, W., Feng, Z., Zon, L.I., and Armstrong, S.A. (2010). The Wnt/beta-catenin pathway is required for the development of leukemia stem cells in AML. Science *327*, 1650–1653.

Welch, J.S. (2018). Patterns of mutations in TP53 mutated AML. Best Pract. Res. Clin. Haematol. *31*, 379–383. https://doi.org/10.1016/j.beha.2018.09.010.

Willert, K., Brown, J.D., Danenberg, E., Duncan, A.W., Weissman, I.L., Reya, T., Yates, J.R., III, and Nusse, R. (2003). Wnt proteins are lipid-modified and can act as stem cell growth factors. Nature *423*, 448–452.

Wu, A., and Jiang, X. (2022). p21-Activated kinases as promising therapeutic targets in hematological malignancies. Leukemia *36*, 315–326. https://doi.org/10.1038/s41375-021-01451-7.

Yeung, J., Esposito, M.T., Gandillet, A., Zeisig, B.B., Griessinger, E., Bonnet, D., and So, C.W.E. (2010). beta-Catenin mediates the establishment and drug resistance of MLL leukemic stem cells. Cancer Cell *18*, 606–618.

Zeng, Y., Broxmeyer, H.E., Staser, K., Chitteti, B.R., Park, S.J., Hahn, S., Cooper, S., Sun, Z., Jiang, L., Yang, X., et al. (2015). Pak2 regulates hematopoietic progenitor cell proliferation, survival, and differentiation. Stem Cell. *33*, 1630–1641. https://doi.org/10.1002/stem.1951.

Zhang, H., Wang, Z., Zhang, J., Li, Z., Liu, J., Yu, J., Zhao, Y., Guo, F., Chen, W.D., and Wang, Y.D. (2024). A MYC-STAMBPL1-TOE1 positive feedback loop mediates EGFR stability in hepatocellular carcinoma. Cell Rep. *43*, 114812. https://doi.org/10.1016/j.celrep.2024.114812.

Zhao, Z.S., and Manser, E. (2012). PAK family kinases: Physiological roles and regulation. Cell. Logist. *2*, 59–68. https://doi.org/10.4161/cl.21912.

Stem Cell Reports, Volume *21*

Supplemental Information

TOE1 is a β-catenin interacting protein regulating the proliferation of hematopoietic cells through PAK2 modulation

Hyun Park, Okan Sevim, Megan Wagstaff, Aaron Goff, David A. Palmer, Bomee Kim, Kate Heesom, Allison Blair, Sarah F. Newbury, Ethan L. Morgan, Benjamin P. Towler, Timothy J. Chevassut, and Rhys G. Morgan

Supplementary Table S2. Forward and reverse primer sequences utilised for RT-qPCR.

Oligo name	Sequence (5'→3')
GAPDH (Forward)	ACAGTCAGCCGCATCTTCTT
GAPDH (Reverse)	ACGACCAAATCCGTTGACTC
LEF1 (Forward)	AGAACACCCCGATGACGG
LEF1 (Reverse)	GGCATCATTATGTACCCGGAAT
PAK2 (Forward)	TGAGCACACCATCCATGTTGG
PAK2 (Reverse)	AGGTCTGTAGTAATCGAGCCC

Supplementary Table S3. Antibodies utilised for immunoblotting, immunofluorescence and flow cytometric assessment.

Antibody	Species	Manufacturer	Cat. number	Dilution
GAPDH	Mouse	Proteintech	60004-1-Ig	1:50,000
β-actin	Mouse	Merck Millipore	A1978	1:50,000
α-Tubulin	Mouse	Merck Millipore	T9026	1:50,000
Lamin A/C	Mouse	Merck Millipore	SAB4200236	1:50,000
β-catenin	Mouse	BD Biosciences	610154	1:2,000
TOE1	Rabbit	Bethyl	A303-643A	1:1,000
LEF-1	Rabbit	Cell Signalling Technology	2230S	1:1,000
TCF4	Rabbit	Cell Signalling Technology	2569S	1:1,000
TCF1	Rabbit	Cell Signalling Technology	2203S	1:1,000
PAK2	Rabbit	Cell Signalling Technology	2608S	1:1,000
PARN	Rabbit	Abcam	AB188333	1:1,000
CD34-PE	Mouse	BioLegend	343505	1:100
PE Mouse IgG1, κ Isotype Ctrl Antibody	Mouse	BioLegend	400111	1:100
CD45-PerCPCy5.5	Mouse	BioLegend	393409	1:100
PerCP/Cy5.5 Mouse IgG1, κ	Mouse	Biolegend	400149	1/100

Isotype Ctrl Antibody				
CD36-PE	Mouse	BioLegend	336205	1:100
PE Mouse IgG2a, κ Isotype Ctrl (FC) Antibody	Mouse	BioLegend	400213	1:100
CD13-PerCPCy5.5	Mouse	BioLegend	301713	1:100
PerCPCy5.5 IgG1 isotype control	Mouse	BioLegend	260114	1:100
PerCP/Cyanine5.5 Mouse IgG1, κ Isotype Ctrl	Mouse	BioLegend	400149	1:100
Alexa Fluor 647 anti-Rabbit IgG	Goat	Invitrogen	A-21245	1:500
Alexa Fluor 488 anti-Mouse IgG	Goat	Invitrogen	A-11001	1:500
anti-Mouse IgG (H+L) Secondary Antibody, HRP	Goat	Invitrogen	A4416	1:1000
anti-Rabbit IgG (H+L) Secondary Antibody, HRP	Goat	Invitrogen	A6154	1:1000

Supplementary Table S4. Lentiviral plasmid profiles utilised for transgene expression.

Target gene	Expression type	Vector type	Source
Non-targeting	shRNA control	pLKO.1-puro Non-Mammalian shRNA Control Plasmid SHC002	Merck Millipore
CTNNB1	shRNA#1	TRCN0000314920	Merck Millipore
CTNNB1	shRNA#2	TRCN0000314921	Merck Millipore
TOE1	shRNA#1	TRCN0000427713	Merck Millipore
TOE1	shRNA#2	TRCN0000152877	Merck Millipore
PAK2	shRNA#1	TRCN0000194671	Merck Millipore
PAK2	shRNA#2	TRCN0000002118	Merck Millipore
PAK2	shRNA#3	TRCN0000002116	Merck Millipore
PAK2	shRNA#4	TRCN0000002115	Merck Millipore
Non-targeting	shRNA control	pLV[shRNA]-EGFP-U6>Scramble (Vector Builder ID:VB230321-1431mhe)	Vector Builder
TOE1	shRNA	pLV[shRNA]-EGFPU6> hTOE1 (Vector Builder ID: VB240320-1219wyt)	Vector Builder
Empty vector control	Ectopic	pLV-EGFP-T2A-Puro-EF1A (Vector Builder ID: VB160723-1006snj)	Vector Builder
TOE1	Ectopic	pLV1-EF1A-hTOE1-Puro (Vector Bulder ID: VB250710-1214jbm)	Vector Builder
PAK2	Ectopic	pLV[Exp]-EGFP/Puro-EF1A>hPAK2 (Vector Builder ID: VB900173-3528fse)	Vector Builder

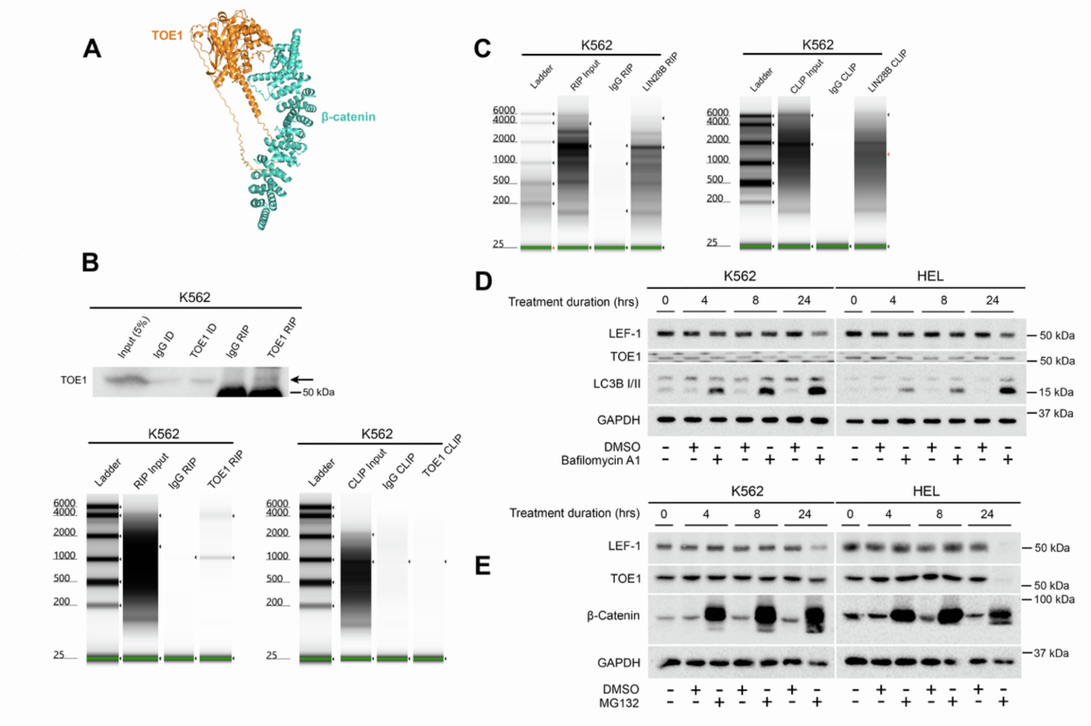

Supplemental Figure S1. β-Catenin forms an RNA-independent interaction with TOE1 in myeloid cells. **(A)** AlphaFold 3 prediction of the β-catenin:TOE1 interaction. The predicted template modelling (pTM) and interface PTM (iPTM) scores were low (0.46 and 0.41 respectively) (Evans et al., 2022; Jumper et al., 2021). To further study the macromolecular interface between the proteins, we utilised PDBePISA (Krissinel and Henrick, 2007). Although there was a considerable hydrophobic interface, as denoted by a solvation free energy gain on formation of the interface ($\Delta^{i}G$) score of -20.8, the complex formation significance score of 0.0 highlighted that the interface is unlikely to be critical for assembly formation. β-Catenin is represented in cyan, whilst TOE1 is represented in orange. **(B)** Immunoblot showing the enrichment of TOE1 in RIP samples above the IgG control. Non-specific bands above and below the TOE1 band (57 kDa) were observed in the co-IP analysis, with the specific TOE1 band represented by an arrow. TapeStation gel images showing the level of RNA isolated following TOE1 immunoprecipitation in RIP and CLIP assays. **(C)** TapeStation gel images demonstrating the enrichment of RNA isolated following LIN28B immunoprecipitation above IgG control in RIP and CLIP assays. Representative immunoblots demonstrating protein levels of LEF-1, β-catenin,

TOE1 and LC3B I/II in K562 and HEL cells ± **(D)** 1μM MG132 or **(E)** 100nM Bafilomycin A1. GAPDH was utilised as the loading control.

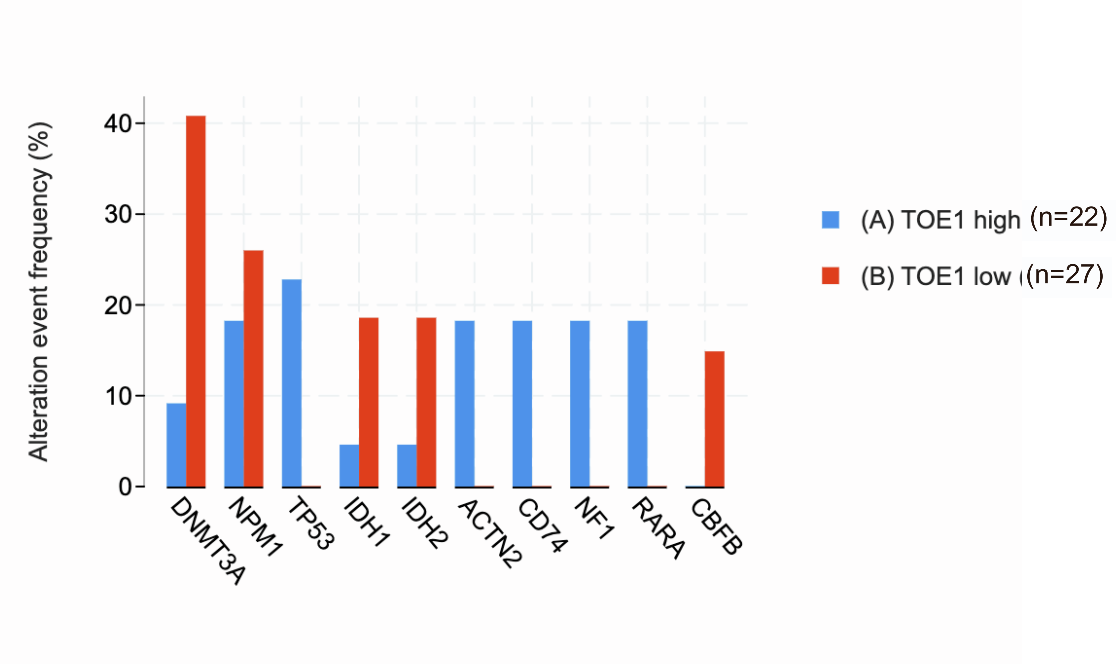

Supplemental Figure S2. *TP53* mutations are enriched in TOE1^{high} samples. Summary graph highlighting the top 10 most frequently altered genes in TOE1^{high} (z score ≥1; n=22) versus TOE1^{low} (z-score <1; n=27) mRNA samples, as derived from the New England Journal of Medicine (NEJM) 2013 The Cancer Genome Atlas (TCGA) acute myeloid leukemia whole exome sequencing data set.

Supplemental Table S5. Altered genes significantly enriched in TOE1^{high} AML patients as deduced from a two-sided Fished exact test.

Gene	Cytoband	% of patients with altered gene	p-Value
TP53	17p13.1	22.73% (5/22)	0.0138
ACTN2	1q43	18.18% (4/22)	0.0345
CD74	5q33.1	18.18% (4/22)	0.0345
NF1	17q11.2	18.18% (4/22)	0.0345

RARA	17q21.2	18.18% (4/22)	0.0345

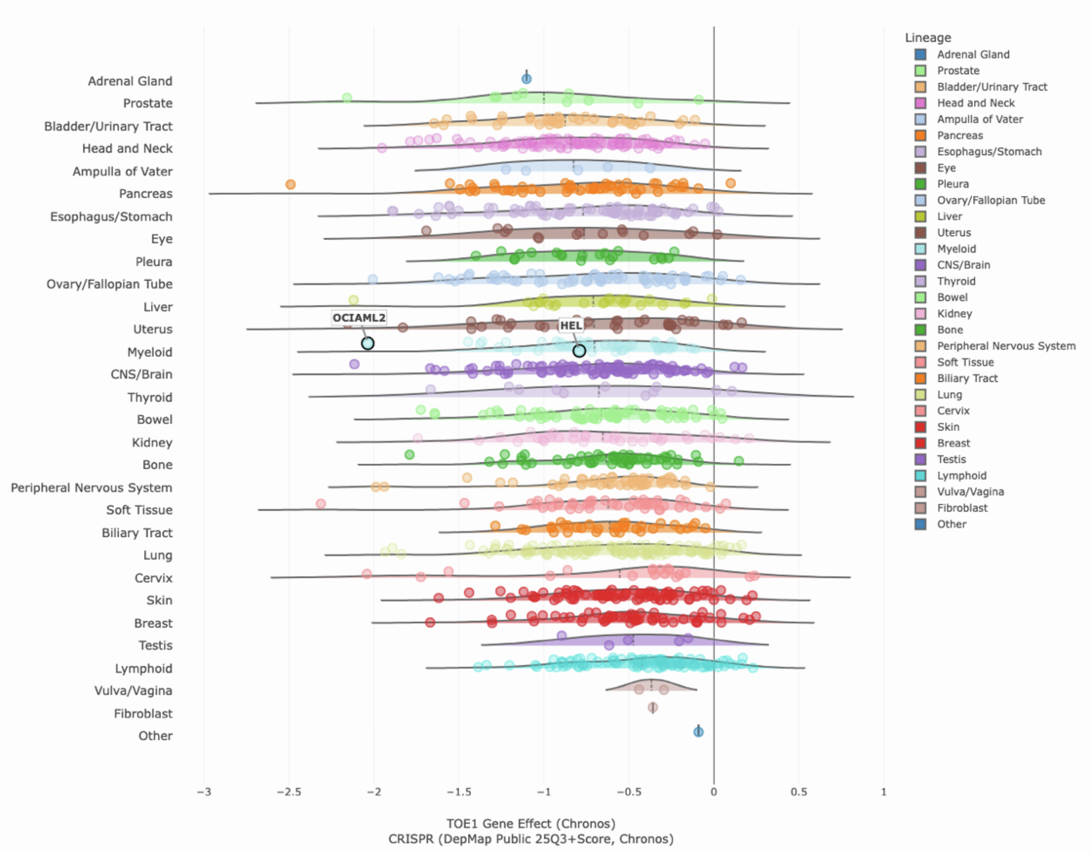

Supplemental Figure S3. Dependency profiles of TOE1 across a panel of cancer cell lines. Box and whisker plots representing Chronos dependency scores (based on cell depletion assays) of cancer cell lines derived from the DepMap CRISPR 23Q2+ data set(Institute, 2021). Numbers of cell lines within each lineage are shown in brackets. A Chronos score of -1 depicts the median of all pan-essential genes (represented as a red line). Lower Chronos scores represent a higher likelihood of *TOE1* dependency demonstrated by the cell line. OCI-AML2 and HEL positions are marked with Gene Effect (Chronos) scores of -2.01 and -0.791, respectively.

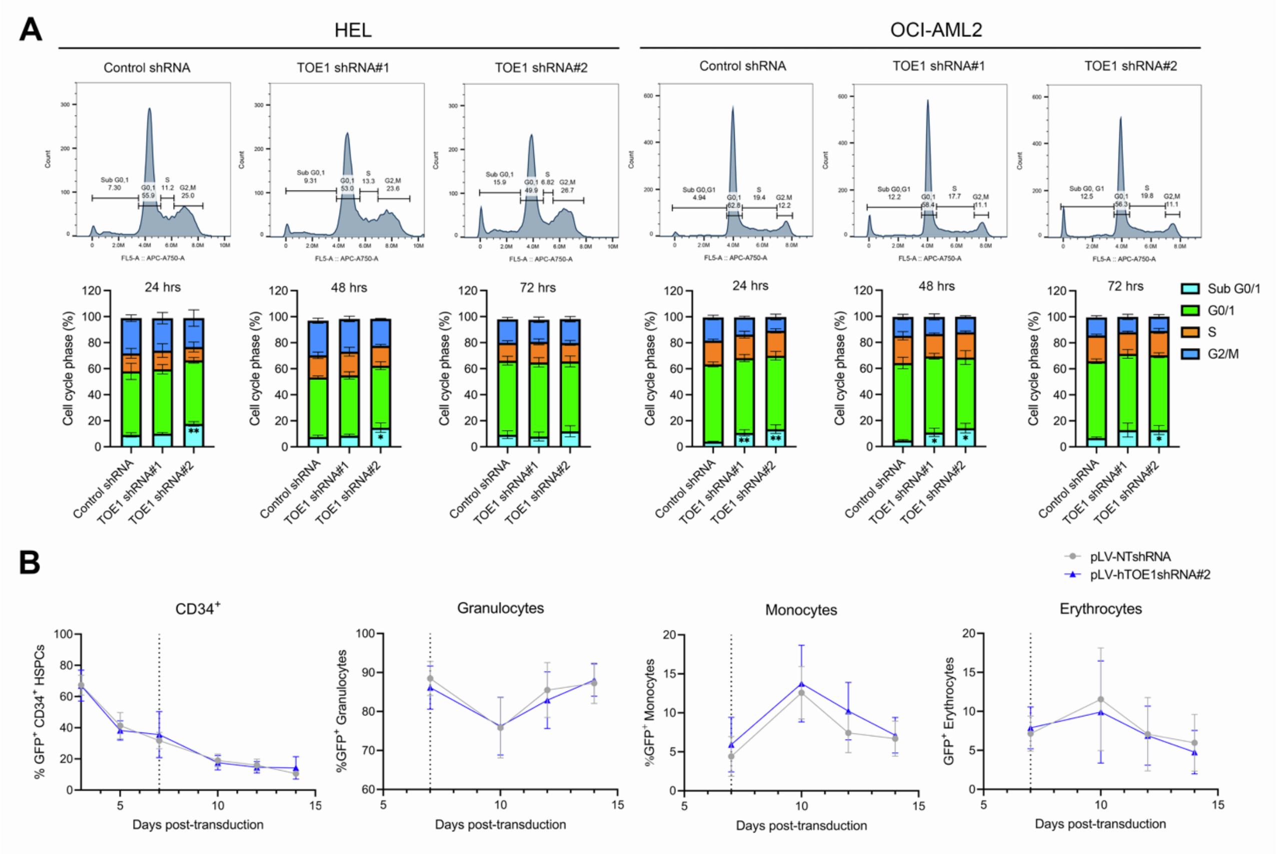

Supplemental Figure S4. TOE1 does not impact cell cycle status or myeloid differentiation in haematopoietic cells. (A) Representative flow cytometric DNA histograms demonstrating cell cycle stages at 48 hours in culture and summary graphs depicting the proportion (%) of cells within each of the sub-G0/G1, G0/1, S and G2/M phases represented over a period of 72 hours in culture in HEL and OCI-AML2 cells (n=3). **(B)** Vertical line at day 7 post-transduction represents initiation of steady state differentiation in CD34$^+$ HSPCs. Lineage discrimination markers were utilised to quantify granulocytic, monocytic and erythrocytic populations utilising flow cytometry (n=3). Error bars indicate mean ± 1SD. Statistical significance is denoted as *p<0.05, **p<0.01 (Student's *t*-test).

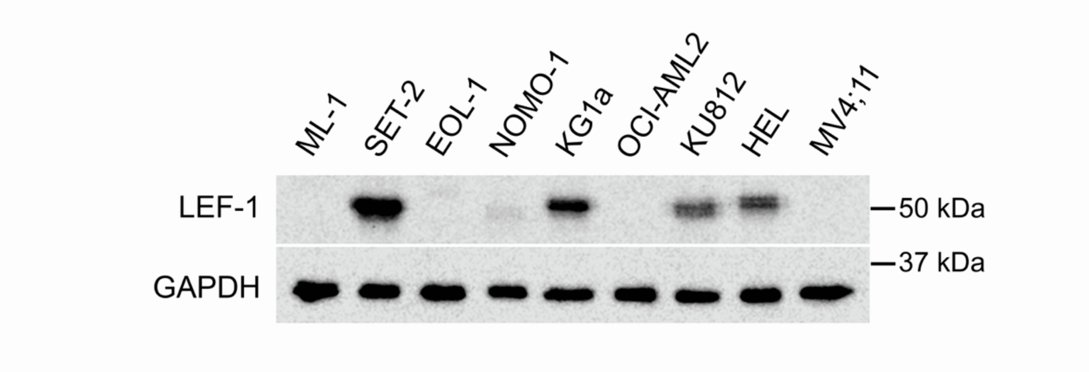

Supplemental Figure S5. Immunoblot demonstrating the protein expression of LEF-1 across a panel of myeloid cell lines. GAPDH was utilised as the loading control.

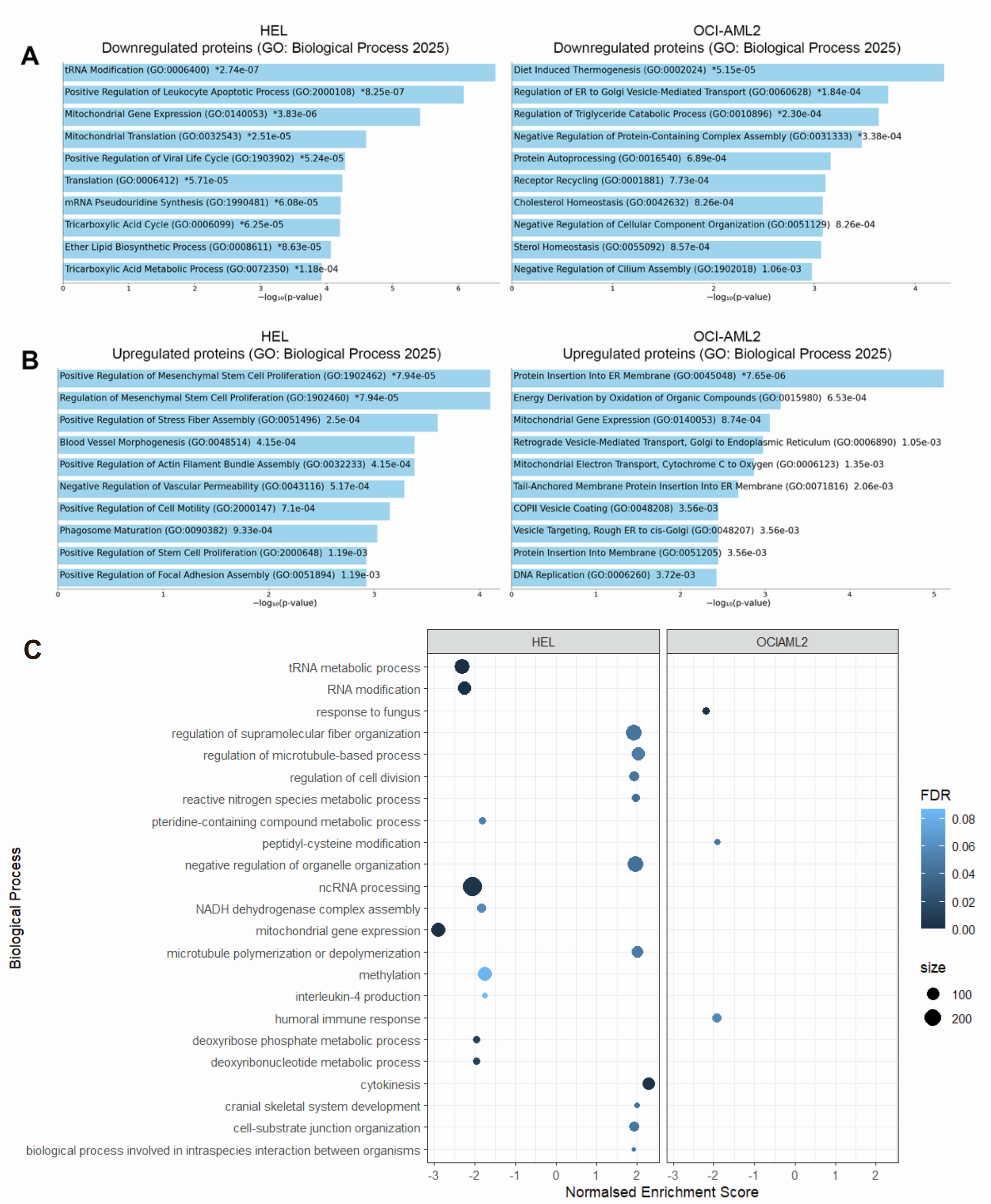

Supplemental Figure S6. Biological Process annotations of enriched proteins in mass spectrometry analysis. Gene ontology overrepresented terms associated with significantly **(A)** downregulated proteins and significantly **(B)** upregulated proteins in response to TOE1 depletion are shown. Statistical significance, defined by adjusted p-values <0.05 derived from Benjamini-Hochberg procedure, are highlighted with an asterix (*). **(C)**

TOE1 regulates the proliferation of hematopoietic cells
Park H, Sevim O et al, Stem Cell Reports, 2026
Supplementary information
Gene set enrichment analysis of HEL and OCI-AML2 cell lines showing 'Biological Processes' significantly altered by TOE1 depletion.

Supplemental Table S6. Common significantly downregulated peptides associated with TOE1 knockdown in myeloid cells.

Accession number	Protein name	Gene name	HEL abundance ratio	OCI-AML2 abundance ratio	HEL Adjusted P-value	OCI-AML2 Adjusted P-value
Q5EBM0	UMP-CMP kinase 2, mitochondrial	CMPK2	0.545	0.774	0.00298543	0.04657373
Q14764	Major vault protein	MVP	0.55	0.866	0.02459156	0.04157605
Q14651	Plastin-1	PLS-1	0.572	0.532	0.00664552	0.04445875
Q96PZ0	Pseudouridylate synthase 7 homolog	PUS-7	0.619	0.717	0.00160676	0.02206208
Q13177	Serine/threonine-protein kinase PAK 2	PAK2	0.629	0.615	0.01342231	0.02284624
C9JG97	Angio associated migratory cell protein	AAMP	0.703	0.949	0.04527474	0.00841372
D6RHI9	Ribonuclease T2	RNASE T2	0.765	0.826	0.0289509	0.04961872
A0A994J7E5	Centrosomal protein of 97 kDa	CEP97	0.796	0.782	0.04982668	0.00811732
P53602	Diphosphomevalonate decarboxylase	MVD	0.84	0.862	0.00228305	0.0207019

Supplementary Table S7. Common significantly upregulated peptides associated with TOE1 knockdown in myeloid cells.

Accession number	Protein name	Gene name	HEL abundance ratio	OCI-AML2 abundance ratio	HEL Adjusted P-value	OCI-AML2 Adjusted P-value
Q8WVM8	Sec1 family domain-containing protein 1	SCFD1	1.087	1.324	0.0359945	0.04336524
P50416	Carnitine O-palmitoyltransferase 1, liver isoform	CPT1A	1.137	1.294	0.0025737	0.00805737
Q969V3	BOS complex subunit NCLN	NCLN	1.161	1.229	0.024636	0.01773498
Q8IWB9	Testis-expressed protein 2	TEX2	1.388	1.123	0.01967537	0.03053523
Q96ME1	F-box/LRR-repeat protein 18	FBXL18	1.392	1.133	0.01684191	0.04806705
Q71UI9	Histone H2A.V	H2AZ2	1.537	1.774	0.03259417	0.03961278
E9PFN4	Anion exchange protein	SLC4A7	1.832	1.462	0.03771812	0.03188565
Q99523	Sortilin	SORT1	2.007	1.567	0.00590551	0.04473766

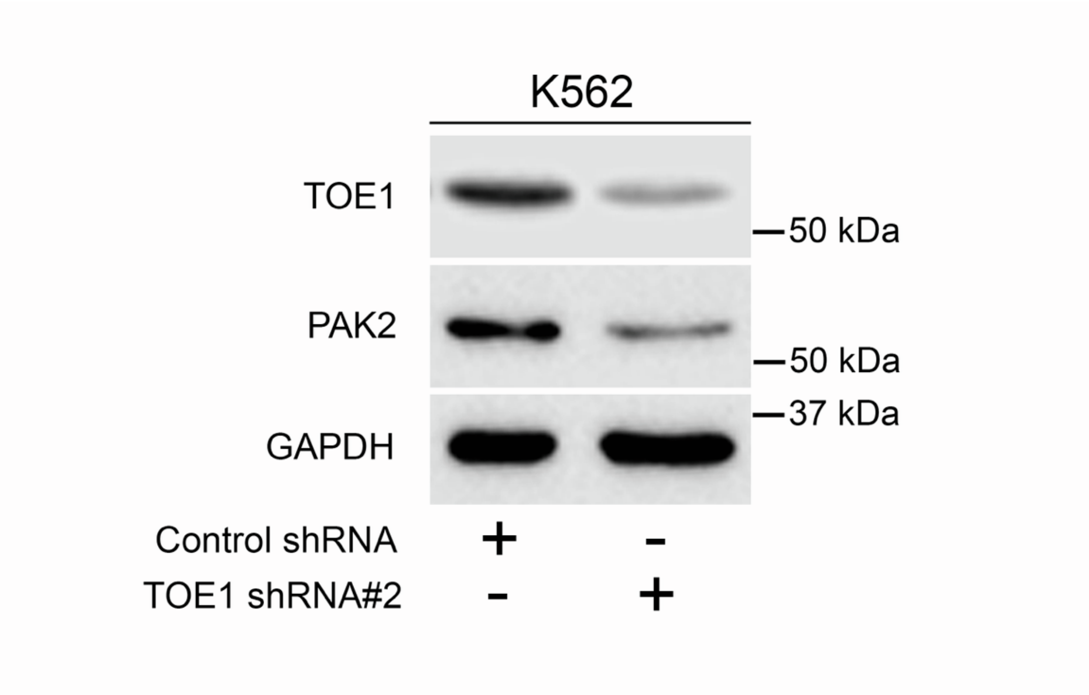

Supplemental Figure S7. Immunoblot demonstrating the protein expression of PAK2 in TOE1 depleted K562 cells. GAPDH was utilised as the loading control.

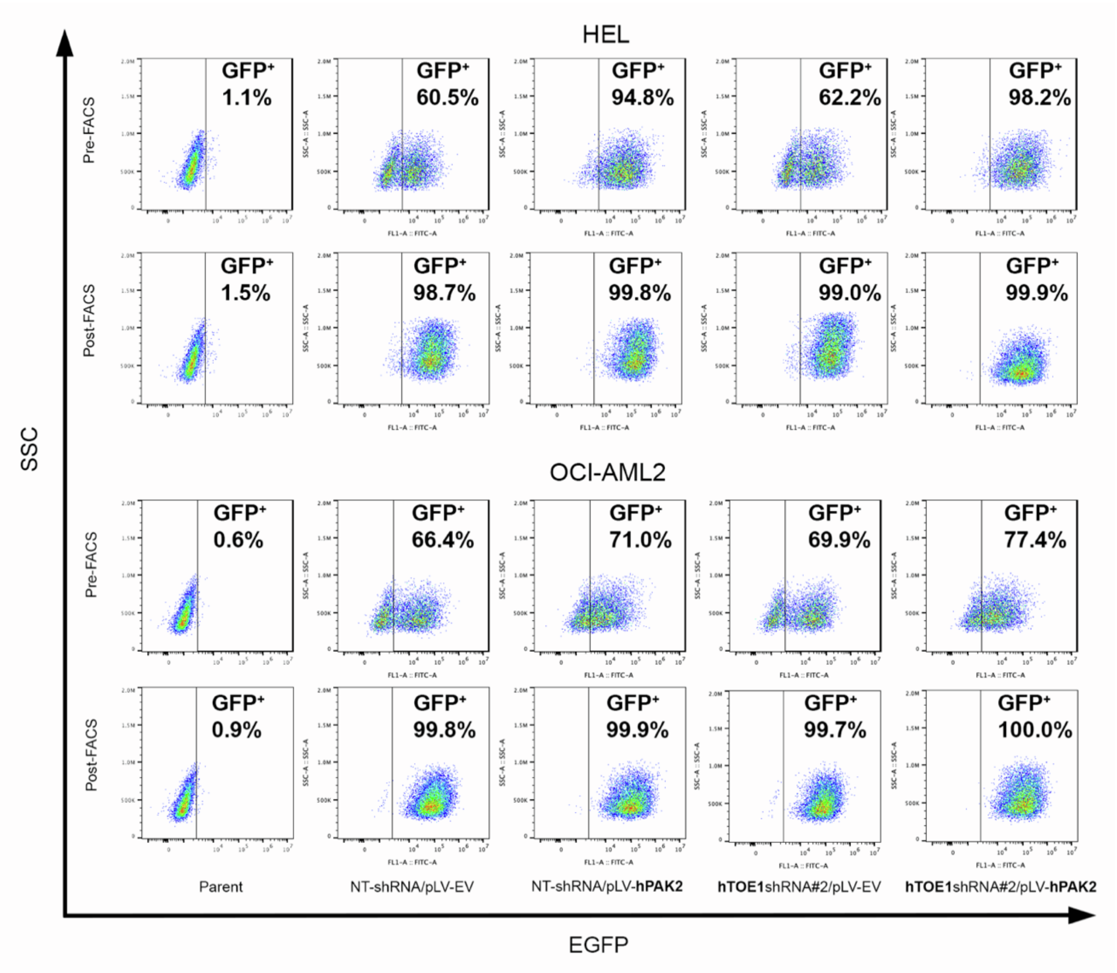

Supplemental Figure S8. Assessment of GFP+ in lentivirally transduced myeloid leukaemia cells. Flow cytometric plots showing the proportion of enhanced green fluorescent protein (EGFP) cells pre- and post-FACS sorting as an assessment of lentiviral transduction efficiency. The negative EGFP threshold was determined utilising untransduced HEL and OCI-AML2 cells.

TOE1 regulates the proliferation of hematopoietic cells
Park H, Sevim O et al, Stem Cell Reports, 2026
Supplementary information
Supplementary methods

Primary samples

Mononuclear cells (MNCs) with viability >80% following isolation via density gradient separation using Ficoll-Hypaque (Merck-Millipore, Gillingham, Dorset) were included in the study and cryopreserved in liquid nitrogen until experimental use. The $CD34^+$ HSPC fraction was enriched to >80% purity as previously described(Morgan et al., 2013), from cryopreserved cord blood MNC preparations using MiniMACS CD34 microbeads (Miltenyi Biotec, Woking, Surrey) according to the manufacturer's instructions and confirmed by flow cytometric assessment of CD34 positivity.

Cell culture and drug treatments

The myeloid cell lines K562, HL60, HEL, U937, PLB-985, NOMO1, OCI-AML3, EOL-1, ML-1, THP-1, KU812 (The European Collection of Authenticated Cell Cultures) and OCI-AML2, MV4;11, KG1, KG1a SET-2, NB4 and Mono-Mac-6 (Leibniz Institute DSMZ-German Collection of Microorganisms and Cell Cultures GmbH) were confirmed mycoplasma-free (Lonza, Basel, Switzerland) and authenticated via the Eurofins short-tandem repeat (STR) analytical service prior to project start. All cell lines were cultured in sterile non-coated tissue culture flasks (Sarstedt) with Roswell Park Memorial Institute-1640 media (RPMI; Merck-Millipore, Dorset, UK), supplemented with 2mM L-glutamine (Merck-Millipore) and 100IU/mL penicillin/100μg/mL streptomycin (Merck-Millipore) and maintained at 37°C under a humidified atmosphere of 5% carbon dioxide (CO_2). FBS (Biosera, Cholet, France) was supplemented at 20% for the culture of KG1, KG1a, and SET-2 cell lines, whilst all other cultures were supplemented at 10%. K562 and HEL cells harbouring the β-catenin-activated reporter (BAR) or mutant 'found unresponsive' control (fuBAR) were generated previously (Morgan et al., 2019). All cultures were

maintained at a density between 1-10x10^5/mL and all experiments datasets conducted within 15 passages from cryopreservation removal to limit genetic variation. Purified human CB CD34$^+$ HSPCs were maintained as previously(Wagstaff et al., 2025), at 5x10^5/ml in StemSpan SFEMII (StemCell Technologies, Cambridge, Cambridgeshire) supplemented with human recombinant 150ng/ml FLT3L, 150ng/ml SCF, and 20ng/ml TPO (Proteintech, Manchester, UK) following isolation into *in vitro* liquid culture.

TMT Labelling and High pH reversed-phase chromatography

Aliquots of 50µg of each sample were digested with trypsin (1.25µg trypsin; 37°C, overnight), labelled with Tandem Mass Tag (TMT) six plex reagents according to the manufacturer's protocol (Thermo Fisher Scientific) and the labelled samples pooled. The pooled sample was desalted using a SepPak cartridge according to the manufacturer's instructions (Waters, Milford, Massachusetts, USA). Eluate from the SepPak cartridge was evaporated to dryness and resuspended in buffer A (20 mM ammonium hydroxide, pH 10) prior to fractionation by high pH reversed-phase chromatography using an Ultimate 3000 liquid chromatography system (Thermo Fisher Scientific). In brief, the sample was loaded onto an XBridge BEH C18 Column (130Å, 3.5 µm, 2.1 mm X 150 mm, Waters, UK) in buffer A and peptides eluted with an increasing gradient of buffer B (20 mM Ammonium Hydroxide in acetonitrile, pH 10) from 0-95% over 60 minutes. The resulting fractions (concatenated into 15 in total) were evaporated to dryness and resuspended in 1% formic acid prior to analysis by nano-LC MSMS using an Orbitrap Fusion Lumos mass spectrometer (Thermo Scientific).

Nano-LC Mass Spectrometry

High pH RP fractions were further fractionated using an Ultimate 3000 nano-LC system in line with an Orbitrap Fusion Lumos mass spectrometer (Thermo Scientific). In brief, peptides in 1% (vol/vol) formic acid were injected onto an

Acclaim PepMap C18 nano-trap column (Thermo Scientific). After washing with 0.5% (vol/vol) acetonitrile 0.1% (vol/vol) formic acid peptides were resolved on a 500 mm × 75 μm Acclaim PepMap C18 reverse phase analytical column (Thermo Scientific) over a 150 min organic gradient, using 7 gradient segments (1-6% solvent B over 1min., 6-15% B over 58min., 15-32%B over 58min., 32-40%B over 5min., 40-90%B over 1min., held at 90%B for 6min and then reduced to 1%B over 1min.) with a flow rate of 300 nl min^{-1}. Solvent A was 0.1% formic acid and Solvent B was aqueous 80% acetonitrile in 0.1% formic acid. Peptides were ionized by nano-electrospray ionization at 2.0kV using a stainless-steel emitter with an internal diameter of 30 μm (Thermo Scientific) and a capillary temperature of 300°C. All spectra were acquired using an Orbitrap Fusion Lumos mass spectrometer controlled by Xcalibur 3.0 software (Thermo Scientific) and operated in data-dependent acquisition mode using an SPS-MS3 workflow. FTMS1 spectra were collected at a resolution of 120 000, with an automatic gain control (AGC) target of 400 000 and a max injection time of 100ms. Precursors were filtered with an intensity threshold of 5000, according to charge state (to include charge states 2-7) and with monoisotopic peak determination set to Peptide. Previously interrogated precursors were excluded using a dynamic window (60s +/-10ppm). The MS2 precursors were isolated with a quadrupole isolation window of 0.7m/z. ITMS2 spectra were collected with an AGC target of 10 000, max injection time of 70ms and CID collision energy of 35%. For FTMS3 analysis, the Orbitrap was operated at 30 000 resolution with an AGC target of 50 000 and a max injection time of 105ms. Precursors were fragmented by high energy collision dissociation (HCD) at a normalised collision energy of 60% to ensure maximal TMT reporter ion yield. Synchronous Precursor Selection (SPS) was enabled to include up to 10 MS2 fragment ions in the FTMS3 scan. The mass spectrometry proteomics data have been deposited to the ProteomeXchange Consortium via the PRIDE partner(Perez-Riverol et al., 2025) repository with the dataset identifier PXD070891.

Data Analysis

The raw data files were processed and quantified using Proteome Discoverer software v2.4 (Thermo Scientific) and searched against the UniProt Human database (downloaded January 2025: 83095 entries) using the SEQUEST HT algorithm. Peptide precursor mass tolerance was set at 10ppm, and MS/MS tolerance was set at 0.6Da. Search criteria included oxidation of methionine (+15.995Da), acetylation of the protein N-terminus (+42.011Da), methionine loss from the protein N-terminus (-131.04Da) and methionine loss plus acetylation of the protein N-terminus (-89.03Da) as variable modifications and carbamidomethylation of cysteine (+57.021Da) and the addition of the TMT mass tag (+229.163Da) to peptide N-termini and lysine as fixed modifications. Searches were performed with full tryptic digestion and a maximum of 2 missed cleavages were allowed. The reverse database search option was enabled, and all data was filtered to satisfy false discovery rate (FDR) of 5%.

Lentivirus generation and transduction

Briefly, 25cm^2 TC-treated flasks (Sarstedt) were poly-L-lysine-coated (Merck-Millipore) and seeded with 4x10^6 HEK293T packaging cells overnight targeting 80-90% confluence the following day. HEK293T cells were transfected with 4μg psPAX2 and 2.2μg pSL3 (pMD.2G) lentiviral packaging plasmids (Addgene), along with 2.1μg of the desired transfer vector (**Supplemental Table S4**) using LipofectamineTM 3000 transfection reagent (Thermo Fisher Scientific). Lentiviral harvests were collected at 24- and 48-hours post-transfection and snap frozen in liquid nitrogen followed by storage at -80°C. For myeloid cell line or CD34$^+$ HSPC transduction, target lentiviruses were sedimented to the base of 24-well tissue culture dishes (Thermo Fisher Scientific) coated with 40μg/ml retronectin (Takara Bio, London, UK) at 2,000g for 90 minutes, and 1-5x10^5 cells in log phase growth incubated with viral or

non-viral (selection controls) containing wells overnight. Transduced cells were selected through 1µg/mL puromycin (Merck-Millipore) treatment, and/or GFP enrichment via fluorescence activated cell sorting (FACS), depending on target vector. Upon selection completion (as deduced from non-transduced matched lines), target gene modulation was assessed via immunoblotting or flow cytometric assessment of Green Fluorescent Protein (GFP) positivity.

References

Evans, R., O'Neill, M., Pritzel, A., Antropova, N., Senior, A., Green, T., Žídek, A., Bates, R., Blackwell, S., Yim, J., et al. (2022). Protein complex prediction with AlphaFold-Multimer. bioRxiv, 2021.2010.2004.463034. 10.1101/2021.10.04.463034.

Institute, B. (2021). DepMap Portal https://depmap.org/portal/gene/TOE1?tab=overview.
Jumper, J., Evans, R., Pritzel, A., Green, T., Figurnov, M., Ronneberger, O., Tunyasuvunakool, K., Bates, R., Žídek, A., Potapenko, A., et al. (2021). Highly accurate protein structure prediction with AlphaFold. Nature *596*, 583-589. 10.1038/s41586-021-03819-2.

Krissinel, E., and Henrick, K. (2007). Inference of macromolecular assemblies from crystalline state. J Mol Biol *372*, 774-797. 10.1016/j.jmb.2007.05.022.

Morgan, R.G., Liddiard, K., Pearn, L., Pumford, S.L., Burnett, A.K., Darley, R.L., and Tonks, A. (2013). gamma-Catenin is expressed throughout normal human hematopoietic development and is required for normal PU.1-dependent monocyte differentiation. Leukemia *27*, 2096-2100. 10.1038/leu.2013.96.

Morgan, R.G., Ridsdale, J., Payne, M., Heesom, K.J., Wilson, M.C., Davidson, A., Greenhough, A., Davies, S., Williams, A.C., Blair, A., et al. (2019). LEF-1 drives aberrant beta-catenin nuclear localization in myeloid leukemia cells. Haematologica *104*, 1365-1377. 10.3324/haematol.2018.202846.

Perez-Riverol, Y., Bandla, C., Kundu, D.J., Kamatchinathan, S., Bai, J., Hewapathirana, S., John, N.S., Prakash, A., Walzer, M., Wang, S., and Vizcaino, J.A. (2025). The PRIDE database at 20 years: 2025 update. Nucleic Acids Res *53*, D543-D553. 10.1093/nar/gkae1011.

TOE1 regulates the proliferation of hematopoietic cells
Park H, Sevim O et al, Stem Cell Reports, 2026
Supplementary information

Wagstaff, M., Sevim, O., Goff, A., Raynor, M., Park, H., Mancini, E.J., Nguyen, D.T.T., Chevassut, T., Blair, A., Castellano, L., et al. (2025). β-Catenin interacts with canonical RBPs including MSI2 to associate with a Wnt signalling mRNA network in myeloid leukaemia cells. Oncogene. 10.1038/s41388-025-03415-y.
